# The Mechanism of Rh(I)-Catalyzed Coupling of Benzotriazoles and Allenes Revisited: Substrate Inhibition, Proton Shuttling, and the Role of Cationic vs Neutral Species

**DOI:** 10.1021/jacs.4c02679

**Published:** 2024-04-22

**Authors:** Nora Jannsen, Fabian Reiß, Hans-Joachim Drexler, Katharina Konieczny, Torsten Beweries, Detlef Heller

**Affiliations:** Leibniz-Institut für Katalyse e.V., Albert-Einstein-Str. 29a, Rostock 18059, Germany

## Abstract

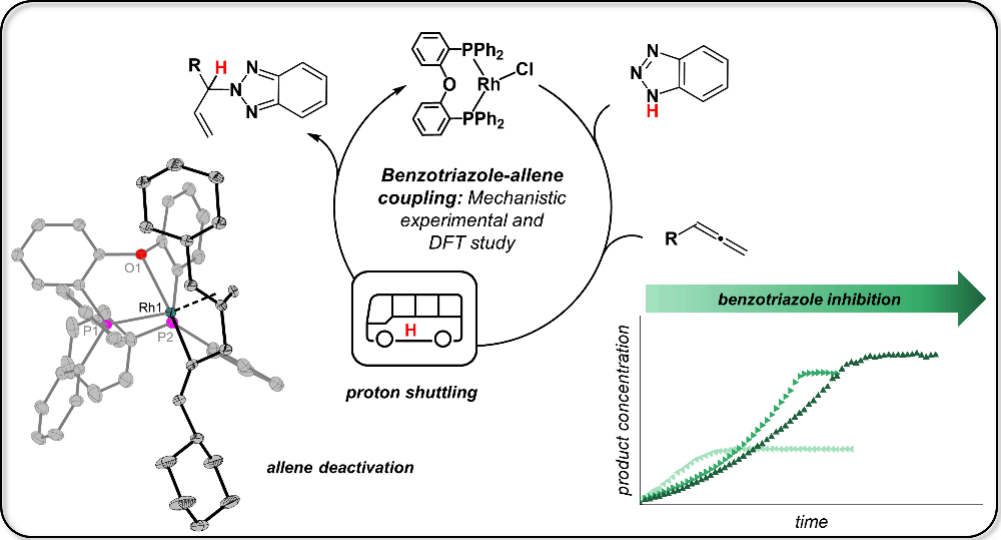

Direct coupling of benzotriazole to unsaturated substrates such as allenes represents an atom-efficient method for the construction of biologically and pharmaceutically interesting functional structures. In this work, the mechanism of the *N*^2^-selective Rh complex-catalyzed coupling of benzotriazoles to allenes was investigated in depth using a combination of experimental and theoretical techniques. Substrate coordination, inhibition, and catalyst deactivation was probed in reactions of the neutral and cationic catalyst precursors [Rh(μ-Cl)(DPEPhos)]_2_ and [Rh(DPEPhos)(MeOH)_2_]^+^ with benzotriazole and allene, giving coordination, or coupling of the substrates. Formation of a rhodacycle, formed by unprecedented 1,2-coupling of allenes, is responsible for catalyst deactivation. Experimental and computational data suggest that cationic species, formed either by abstraction of the chloride ligand or used directly, are relevant for catalysis. Isomerization of benzotriazole and cleavage of its N–H bond are suggested to occur by counteranion-assisted proton shuttling. This contrasts with a previously proposed scenario in which oxidative N–H addition at Rh is one of the key steps. Based on the mechanistic analysis, the catalytic coupling reaction could be optimized, leading to lower reaction temperature and shorter reaction times compared to the literature.

## Introduction

**Figure 1:**
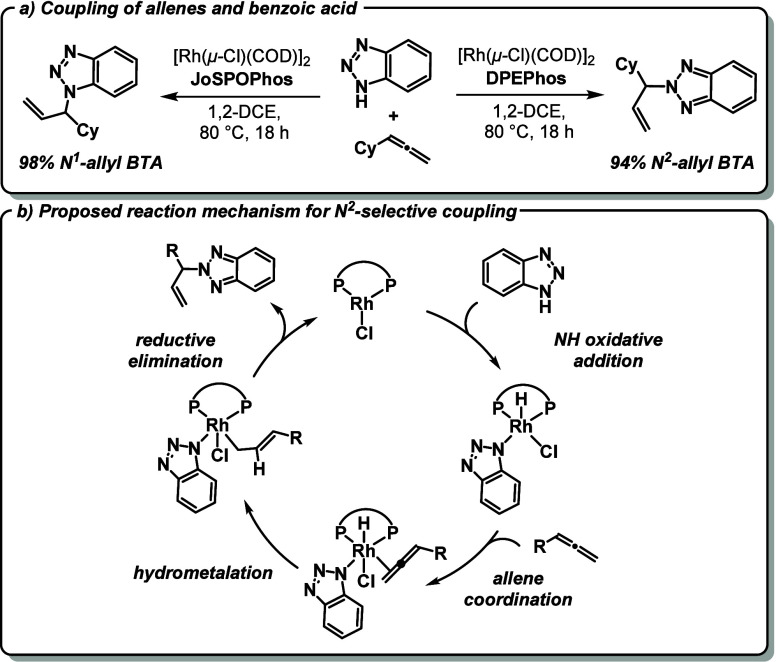
(a) Rh complex-catalyzed coupling of BTAH and allenes with *N*^1^- and *N*^2^-selectivity.^1^ (b) Proposed mechanism of the *N*^2^-selective reaction.^1,13^

The broad spectrum of biological activities makes *N*-alkyl benzotriazole derivatives valuable building blocks in pharmaceutical and medicinal chemistry.^1^ For example, *N*-alkyl-substituted benzotriazoles can have anti-inflammatory,^2^ antifungal,^3^ antibacterial,^4^ analgesic,^5^ and antidepressant^5^ effects. Benzotriazole (BTAH) has an *N*^1^- and an *N*^2^-tautomer, the first of which is energetically preferred due to its fully aromatic nature.^6−10^ As a result, the selective *N*^2^-substitution is challenging and mixtures of *N*^1^- and *N*^2^-substituted benzotriazole derivatives are often obtained. The first example of a rhodium-catalyzed coupling of benzotriazoles and allenes was published in 2014 by Breit *et al*.^11^ The key aspect of this atom economic reaction represents the high *N*^1^- or *N*^2^-selectivity, which can be controlled through the choice of the diphosphine ligand (Figure 1a).

While reactions using the phosphine-oxide-based chiral JoSPOPhos^12^ ligand almost exclusively produce *N*^1^-allyl benzotriazole (*N*^1^:*N*^2^ = 98:2), use of the achiral DPEPhos ligand reverts the selectivity and mainly gives the *N*^2^-functionalized product (94%). Computational analysis of this remarkable ligand-divergent selectivity suggested a general catalytic cycle consisting of four key mechanistic steps, all involving neutral Rh species (Figure 1b):^13^ (i) N–H oxidative addition of the BTAH to the monomerized precatalyst [Rh(Cl)(diphosphine)], (ii) coordination of the allene, (iii) hydrometalation of the coordinated allene, and (iv) reductive elimination of the *N*-alkylated heterocycle.

Evidence for oxidative addition of BTAH, the proposed first step of the reaction, was found by ^1^H NMR analysis of reactions of the *in situ*-generated precatalyst [Rh(μ-Cl)(DPEPhos)]_2_ with one equivalent of BTAH at −10 °C in CDCl_3_, where a well-defined hydride signal could be observed at δ −14.7 ppm at the onset of the reaction.^1^ Computational studies using activation strain and energy decomposition analyses implied that high *N*^2^-selectivity of the DPEPhos-derived catalyst system originates in a more stabilizing interaction between the electron rich *N*^1^-atom of BTAH and the positively charged Rh in the proposed first reaction step, the oxidative addition.

A detailed experiment-based understanding of the mechanism of this highly interesting coupling reaction, rationalizing catalyst activation, deactivation, and inhibition pathways that account for the comparably high reaction temperature and time (80 °C, 18 h), is, however, still lacking. Specific and in-depth mechanistic analysis of chemical reactions can be very time-consuming and are therefore often neglected.^14^ However, it is the only way to identify activation and deactivation pathways, allowing for fundamental optimization of the respective catalytic process, and providing a basis for accurate density functional theory (DFT) studies to support the corresponding mechanistic proposal.^15−17^ In the present work, we focus on the study of the *N*^2^-selective DPEPhos-based system as this mode of BTAH functionalization is more challenging, aiming at circumventing possible deactivation or inhibition pathways, ultimately resulting in an optimization of the reaction conditions.

## Results and Discussion

### Substrate Coordination

#### Benzotriazole Complexes

**Scheme 1:**
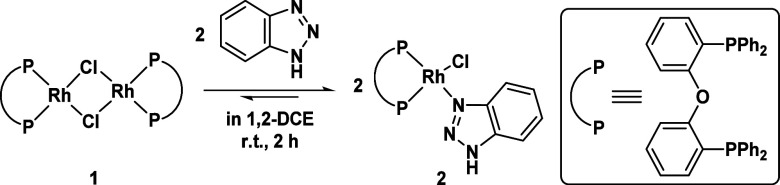
Synthesis of [Rh(Cl)(DPEPhos)(BTAH)] (**2**)

As a starting point, the reactions of both substrates with the precatalyst should be understood in order to further analyze the catalytic reaction in the next step. As mentioned above, first NMR studies indicated an oxidative addition of BTAH to [Rh(μ-Cl)(DPEPhos)]_2_**1** as an initial step of the catalytic cycle. We repeated this experiment using catalytically relevant 1,2-dichloroethane (1,2-DCE) at room temperature (Scheme 1).

**Figure 2:**
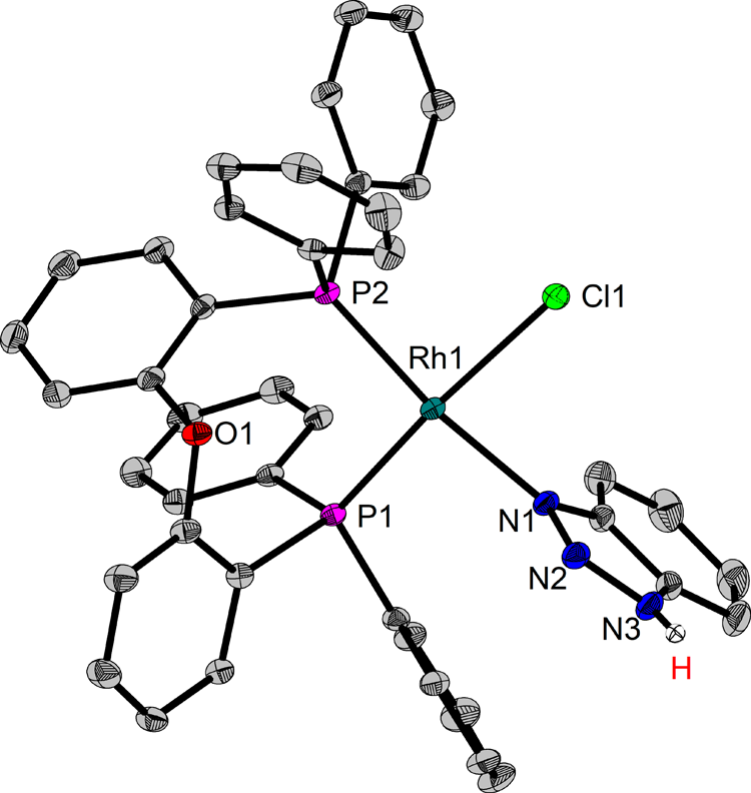
Molecular structure of [Rh(Cl)(DPEPhos)(BTAH)] **2**. Hydrogen atoms (except for NH) are omitted for clarity (ORTEP, 30% probability ellipsoids). Selected bond length and angles: Rh–P 2.2211(6)-2.2384(6) Å, Rh–Cl 2.4034(6) Å, Rh–N 2.096(19) Å, P–Rh–P 96.56(2)°, N1–Rh–Cl1 83.34(6)°.

To our surprise, immediate formation of an amorphous solid occurred, further preventing the NMR spectroscopic analysis. Dissolving the orange precipitate in a mixture of THF/1,2-DCE and layering with *n*-heptane results in the crystallization of orange material suitable for single crystal X-ray diffraction (SC-XRD) analysis. The molecular structure (Figure 2) confirms the reaction between the rhodium species and the heteroarene, but unexpectedly *no* N–H oxidative addition of BTAH occurred. Instead, dative coordination of BTAH via the *N*^3^-atom is found, while the N–H bond remains intact.

**Figure 3:**
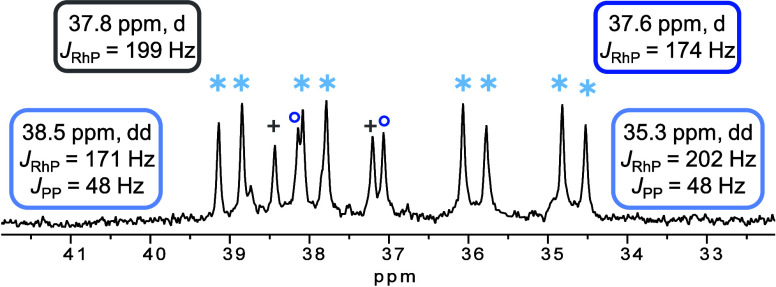
Low temperature ^31^P{^1^H} NMR spectrum (162 MHz, −51 °C) of dissolved crystals of **2** in a mixture of THF-*d*_8_ and 1,2-DCE-*d*_4_.

No Rh hydride signals can be observed in the ^1^H NMR spectrum (Figure S1) of the dissolved crystals, suggesting that this species does not convert into a Rh(III) species through oxidative addition of the precoordinated BTAH. Instead, an N–H signal is present at 14.5 ppm. The ^31^P{^1^H} NMR spectrum (Figure S2) reveals a broad signal at approximately 38 ppm. The origin of the broad signal was investigated in variable temperature NMR experiments (Figure S3) and the corresponding ^31^P{^1^H} spectrum at −51 °C is shown in Figure 3. The two doublets of a doublet (dd) at 35.3 (*J*_RhP_ = 202 Hz, *J*_PP_ = 48 Hz) and 38.5 ppm (*J*_RhP_ = 171 Hz, *J*_PP_ = 48 Hz) can be assigned to crystallographically characterized mono-BTAH complex [Rh(Cl)(DPEPhos)(BTAH)] (**2**).

In a ^31^P{^1^H} COSY NMR experiment both dd show correlation to each other (Figure S4). Besides species **2** (∗), two more signals (+, o) can be identified in the spectrum. The doublet at 37.8 ppm (+, *J*_RhP_ = 199 Hz) can be assigned to the precatalyst **1**, which is in equilibrium with the mono-BTAH complex, while the origin of the second doublet (o, 37.6 ppm, *J*_RhP_ = 174 Hz) is unknown.

**Scheme 2:**
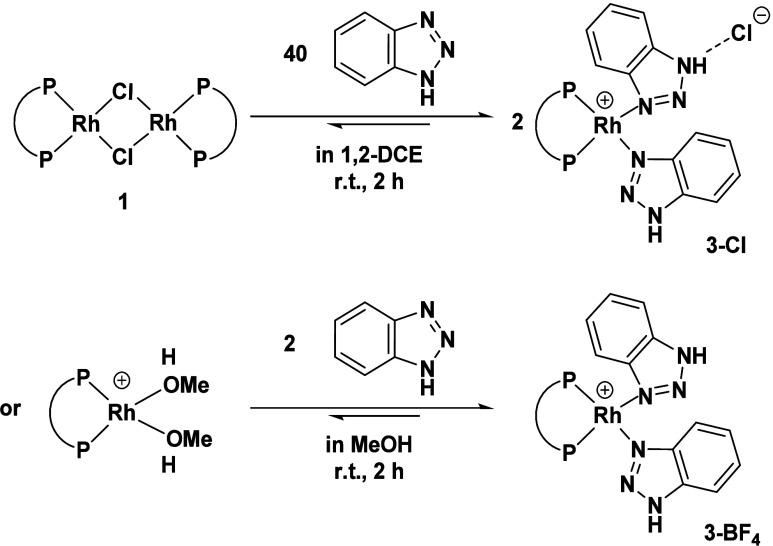
Reactions of Precatalyst **1** and the Cationic Precursor [Rh(DPEPhos)(MeOH)_2_][BF_4_] with an Excess of BTAH

Reaction of **1** with a catalytically relevant increased concentration of BTAH (ratio precatalyst:BTAH = 1:40) at room temperature exclusively furnishes the same species albeit shifted to slightly higher field due to the temperature difference (d, 36.4 ppm, *J*_RhP_ = 179 Hz, Figure 3). The presence of a doublet at 36.4 ppm indicates the formation of a highly symmetric species with chemically equivalent phosphorus atoms. This suggests coordination of two BTAH molecules leading to two possible di-BTAH complexes: a neutral pentacoordinated complex [Rh(Cl)(DPEPhos)(BTAH)_2_] or a cationic species [Rh(DPEPhos)(BTAH)_2_]Cl with a chloride counterion (Scheme 2).

**Figure 4:**
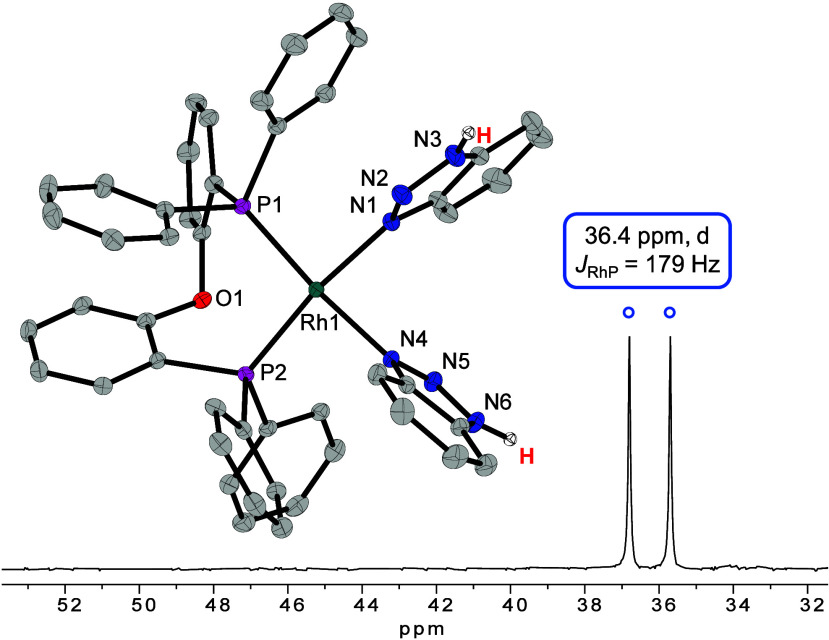
^31^P{^1^H} NMR spectrum (24 °C, 122 MHz) in 1,2-DCE-*d*_4_ of [Rh(DPEPhos)(BTAH)_2_]X (X = Cl in the NMR spectrum and X = [BF_4_] in the molecular structure). Hydrogen atoms (except for NH) and the counterion [BF_4_]^−^ are omitted for clarity (ORTEP, 30% probability ellipsoids). Selected bond length and angles: Rh–P 2.2458(10)-2.2600(11) Å, Rh–N 2.080(3)-2.096(3) Å, P–Rh–P 97.19(4)°, N–Rh–N 82.75(12)°.

Indeed, formation of the latter complex could be confirmed by independent synthesis from the cationic precursor [Rh(DPEPhos)(MeOH)_2_][BF_4_].^18^ The same doublet signal was identified in the ^31^P{^1^H} NMR spectrum, suggesting the decoordination of chloride by BTAH addition (Figure 4). The complex was crystallized from a solution in THF layered with diethyl ether, confirming the formation of the cationic complex [Rh(DPEPhos)(BTAH)_2_]^+^ (**3**) by SC-XRD analysis.

Consequently, the low temperature ^31^P{^1^H} NMR spectrum of the dissolved crystals of **2** (Figure 3) shows that all three species, i.e., precatalyst, mono-BTAH complex, and di-BTAH complex, are in equilibrium. An analogous relationship is known for ammonia coordination to Rh(I) where formation of [Rh(μ-Cl)(diphosphine)]_2_, [Rh(Cl)(diphosphine)(NH_3_)], and [Rh(diphosphine)(NH_3_)_2_]Cl was found before.^19^ The observation of all three species in parallel in only one NMR experiment is however unknown. For BTAH, it could not only be shown that a cationic rhodium complex **3** forms from the neutral precatalyst **1** but also that this is in equilibrium with the neutral complex **2** and the precatalyst. The mechanism of the overall catalytic reaction is likely to be affected by this equilibrium, potentially leading to substrate inhibition due to the formation of inactive species **3**.

#### Allene Complexes

**Figure 5:**
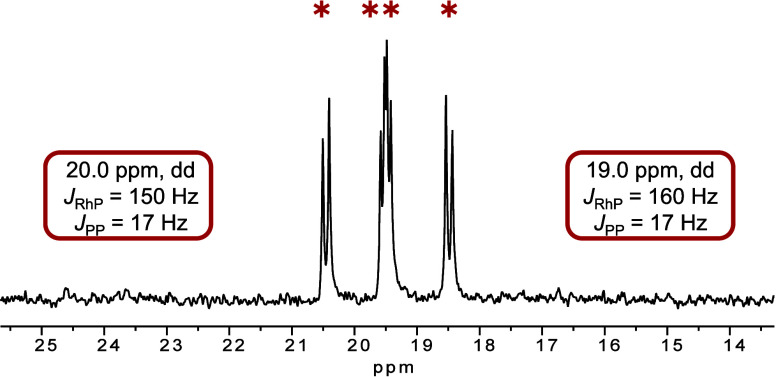
^31^P{^1^H} NMR spectrum (24 °C, 162 MHz) of reaction of [Rh(DPEPhos)(MeOH)_2_][BF_4_] with 2 equiv of cyclohexyl allene in MeOH-*d*_4_.

When precatalyst **1** is reacted with 2 equiv of *n*-pentyl allene or cyclohexyl allene, its characteristic doublet can no longer be detected in the ^31^P{^1^H} NMR spectrum. Instead, two new broad doublets can be observed (Figure S5).^20^ The ^1^H and ^31^P{^1^H} NMR spectra at a molar Rh:allene ratio of 1:1 show full conversion, which indicates the simple coordination of an allene molecule to the 14-electron monomerized catalyst species [Rh(Cl)(DPEPhos)] **1a** and the formation of monoallene complexes of the type [Rh(Cl)(DPEPhos)(allene)] **4**. The broad doublets corresponding to complexes [Rh(Cl)(DPEPhos)(cyclohexyl allene)] (4-cyclohexyl, 26.8 ppm, *J*_RhP_ ≈ 150 Hz; 18.4 ppm, *J*_RhP_ ≈ 155 Hz) and [Rh(Cl)(DPEPhos)(*n*-pentyl allene)] (4-*n*-pentyl, 25.2 ppm, *J*_RhP_ ≈ 158 Hz; 18.3 ppm, *J*_RhP_ ≈ 145 Hz) can be attributed to competitive 1,2- and 2,3-coordination of the allene as already described for other Rh allene complexes.^21^ For both isomers (i.e., 1,2- and 2,3-coordination) of 4-*n*-pentyl, a total stability constant *K*_4-*n*-pentyl, total_ ≈ 31.000 L·mol^–1^ can be determined by a UV–vis spectroscopic titration (Figures S6 and S7).^22^ While the stoichiometric conversion leads to a similar result for both allenes, an increase in the allene concentration shows a very different reactivity. Using 65 equiv of *n*-pentyl allene, the spectrum remains unchanged, and no follow-up reaction could be observed (Figure S8). In contrast, a higher concentration of cyclohexyl allene leads to the formation of a new complex. Already with a molar ratio of 1:7, the signal of the main species appears as two doublets of a doublet in the ^31^P{^1^H} NMR spectrum (Figure S9; dd, 19.0 ppm, *J*_RhP_ = 150 Hz, *J*_PP_ = 17 Hz; dd, 20.0 ppm, *J*_RhP_ = 160 Hz, *J*_PP_ = 17 Hz). Interestingly, the same signals are found when converting the cationic complex [Rh(DPEPhos)(Solv)_2_][BF_4_] (Solv = MeOH, THF) with 2 equiv of the same allene (Figure 5).

**Figure 6:**
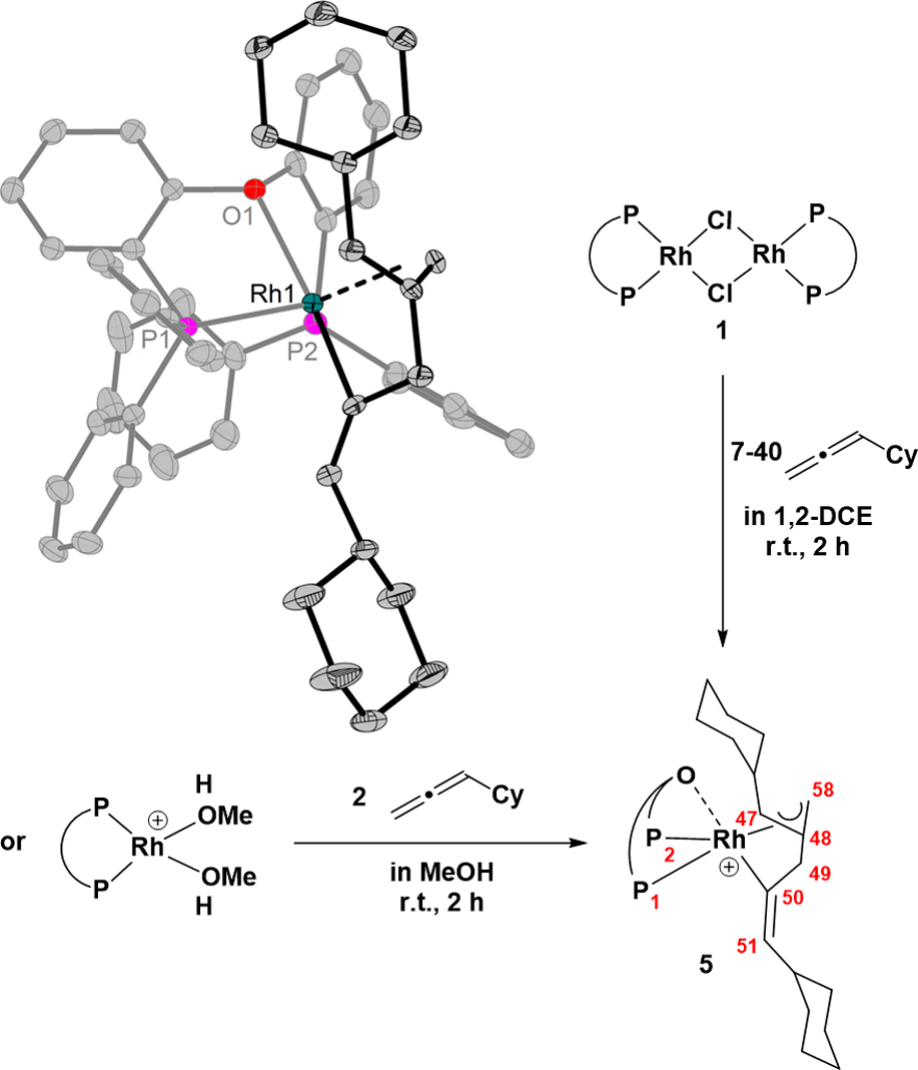
Formation and molecular structure of the rhodacycle (**5**). Hydrogen atoms and the counterion [BF_4_]^−^ are omitted for clarity (ORTEP, 30% probability of ellipsoids).

In the ^1^H NMR spectrum, no unreacted allene is detectable, indicating the conversion of both allene molecules. Colorless needles of this species, suitable for SC-XRD, could be obtained from MeOH solution. The molecular structure (Figure 6) reveals dimerization of the allene to furnish the metallacyclic species instead of simple coordination. This type of reactivity of allenes with transition-metal complexes is unexpected and hitherto unknown.

The six-coordinate 18-electron complex can be best described as a Rh^III^-π-allyl-σ-vinyl-complex **5**. In this metallacycle, the Rh center shows σ*-*coordination to the vinyl carbon (Rh1–C50 2.023(5) Å) of one part of the allene dimer and a coordinative bond to the π*-*allyl group of the second allene fragment. This coordination motif of two 1,2-coupled allenes has not yet been described in the literature. The unsymmetric allyl moiety shows Rh–C distances of 2.245(5) (C58), 2.141(6) (C48), and 2.268(6) Å (C47), while the Rh1–C49 distance is 2.720(6) Å, indicating no Rh–C bond. The C–C distances in the π*-*allyl unit are 1.409(9) Å (C48–C47) and 1.432(10) Å (C48–C58). The newly formed C48–C49 bond shows a distance of 1.533(8) Å, indicating a C–C single bond. The C50–C51 σ-vinyl bond is 1.306(8) Å long, in the range of C–C double bonds. The DPEPhos ligand shows P,O coordination to the Rh center (Rh1–P1 2.2908(14), Rh1–P1 2.3359(17), Rh1–O1 2.348(4) Å).^23^ Thus, the isolated complex represents not only a rare example of an isolated rhodacycle complex^24^ but also of *k*^3^-P,O,P coordination of the DPEPhos ligand at Rh.^25−28^ Both P and two C atoms (C58, C47) of the π-allyl moiety occupy the equatorial positions of the distorted octahedral coordination polyeder, while the vinyl C and the O atom are found in the axial positions (C50–Rh1–O1 175.35(18)°). A computational bond analysis (see the SI for details) confirms the delocalized π-allyl bond as well as the κ^3^-P,O,P coordination of the DPEPhos ligand with a weak dative Rh–O interaction (Wiberg bond index of 0.32). A detailed computational analysis of the binding situation in rhodacycle **5** can be found in the SI.

Further NMR analysis of **5** shows a ^103^Rh NMR shift of 918 ppm, which was assigned to the set of dd in the ^31^P{^1^H} NMR spectrum in an inverse ^103^Rh^31^P{^1^H} HMQC experiment (Figure S10). In a ^1^H^31^P{^1^H} HMBC experiment (Figure S11), correlations between the ^31^P NMR signal and several ^1^H NMR signals could be observed, which could be further assigned to allyl and vinyl moieties in a ^1^H COSY experiment (Figure 2, Figure S12). The same reactivity was observed for the conversion of [Rh(DPEPhos)(MeOH)_2_][BF_4_] with 2 equiv of *n*-pentyl allene, leading to the formation of analogous *n*-pentyl substituted metallacycle (Figures S13 and S14). Compared to the cyclohexyl-substituted rhodacycle **5**, the conformationally dynamic behavior of the *n*-pentyl moiety leads to a broadening of the ^31^P{^1^H} NMR signal.

#### The Catalytic Reaction

**Figure 7:**
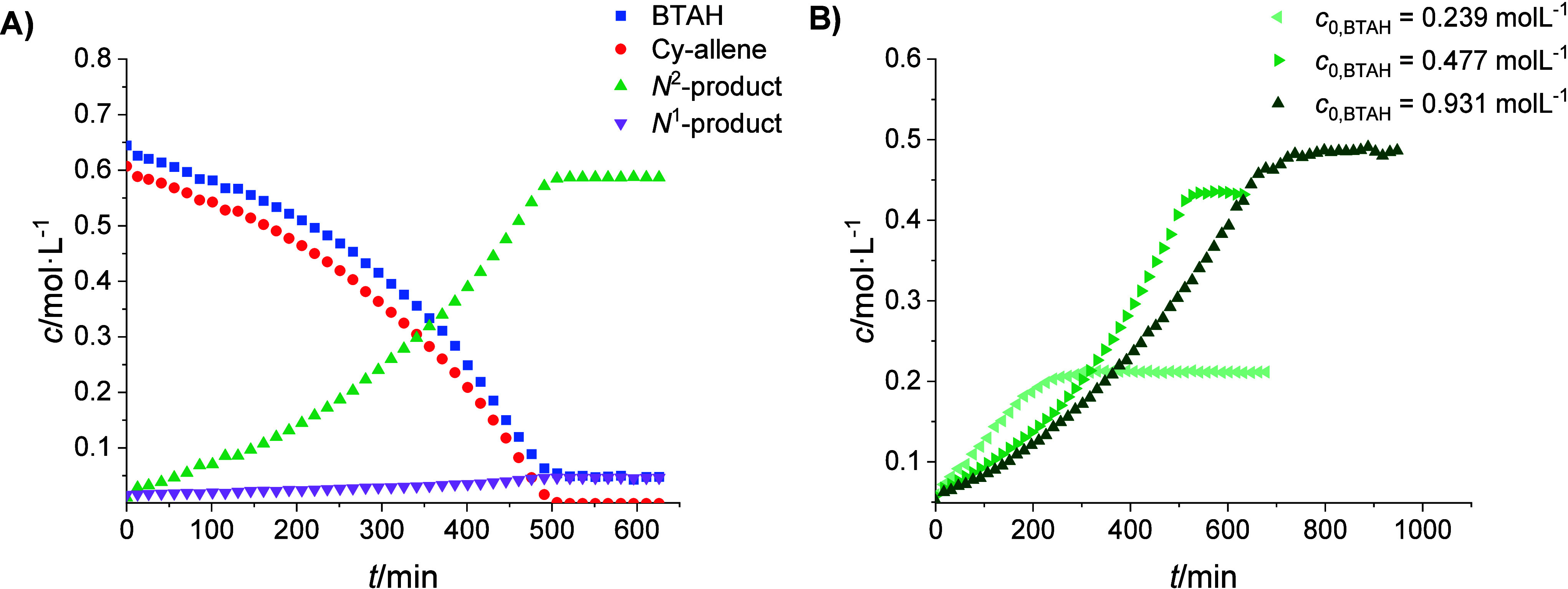
Concentration–time-plots of (A) the conversion of 0.007 mmol precatalyst, 0.4 mmol BTAH and 0.4 mmol cyclohexyl allene in 0.6 mL 1,2-DCE-*d*_4_ monitored by ^1^H NMR spectroscopy and (B) the *N*^2^-product with different BTAH start concentrations *c*_0,BTAH_.

Before reaction monitoring experiments were conducted, the reaction conditions were optimized. The isolated precatalyst [Rh(μ-Cl)(DPEPhos)]_2_**1** was used instead of the *in situ* system ([Rh(μ-Cl](COD)]_2_/DPEPhos) to ensure full conversion to the precatalyst and to exclude negative influences of the free diolefin on the catalysis.^29,30^ It was found that the order of the addition of the substrates has a significant influence on the organometallic species formed during the reaction. If the allene is added first, a high concentration of the above-mentioned Rh(III) rhodacycle **5** was detected, which could be circumvented by first adding BTAH (Figure S15). We could further show that the Rh-catalyzed BTAH-allene coupling proceeds in an acceptable reaction time, even at lower temperatures. Hence, the *in operando* NMR spectroscopic reaction monitoring was performed at 50 °C. The concentration of both reactants and products was calculated from the integrals in the ^1^H NMR spectra (Figure S16). The concentration–time diagram (Figure 7A) shows that the system exhibits a progressive acceleration in the formation of the main product (N2-allyl-BTA) and the consumption of both reactants (BTAH, cyclohexyl-allene), probably due to the slow consumption of a turnover-limiting species. This phenomenon is common for strong substrate inhibition.^31^

To support this assumption and to identify the inhibiting substrate, the BTAH concentration was varied in two different experiments. Indeed, the corresponding experiments indicate a substrate inhibition evoked by BTAH (Figure 7B), giving more pronounced sigmoidal behavior and slower reactions with a higher BTAH concentration. This effect could not be prevented by increasing the starting concentration of the allene substrate (Figure S17). It is, furthermore, remarkable that the reaction at lower substrate concentrations can even be performed at room temperature. If **1** is converted with 20 equiv. BTAH and cyclohexyl allene (each), full conversion to the *N*^2^-product is observed (Figure S18). The reaction with a low Rh:BTAH:allene ratio (1:11:14) was monitored by low-field ^1^H NMR spectroscopy (80 MHz) and is completed after approximately 300 min at room temperature (Figure S19). Of note, the concentration–time plot is not sigmoidal. We therefore conclude that substrate inhibition can be reduced or fully shut down at low substrate concentrations.

The ^31^P{^1^H} NMR spectra (Figure S20) of the reaction monitoring at 50 °C reveal a broad signal at 23–25 ppm that decreases with the progress of the reaction. Two dd at 16.2 ppm (*J*_RhP_ = 131 Hz, *J*_PP_ = 26 Hz) and 32.8 ppm (*J*_RhP_ = 190 Hz, *J*_PP_ = 26 Hz) evolve during the reaction and are the main species at full conversion. This species *cannot* be generated independently by converting the precatalyst with an excess of product (Figure S21). A low temperature NMR experiment at −25 °C, with the aim to sharpen the broad signal (Figure S22, Figure S23) in the beginning of the catalytic reaction, revealed a new dd at 30.3 ppm (*J*_RhP_ = 143 Hz, *J*_PP_ = 30 Hz), which was not observed before and could not be assigned at this stage. In addition, the formation of **5** as a deactivating species could be observed in all reactions. Of note, the higher amount of allene required for the formation of this species starting from the cationic precursor [Rh(DPEPhos)(MeOH)_2_] (Figure 6) could point to a potentially beneficial effect of the chloride, suppressing formation of **5**.

**Scheme 3:**
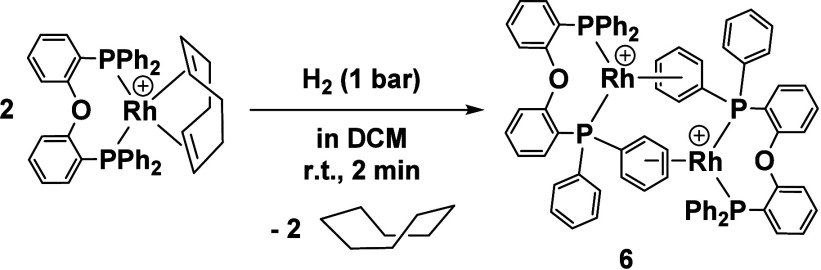
Synthesis of the η^6^-Arene Bridged Dimer **6** The [BF_4_]^−^ anion is omitted for clarity.

**Figure 8:**
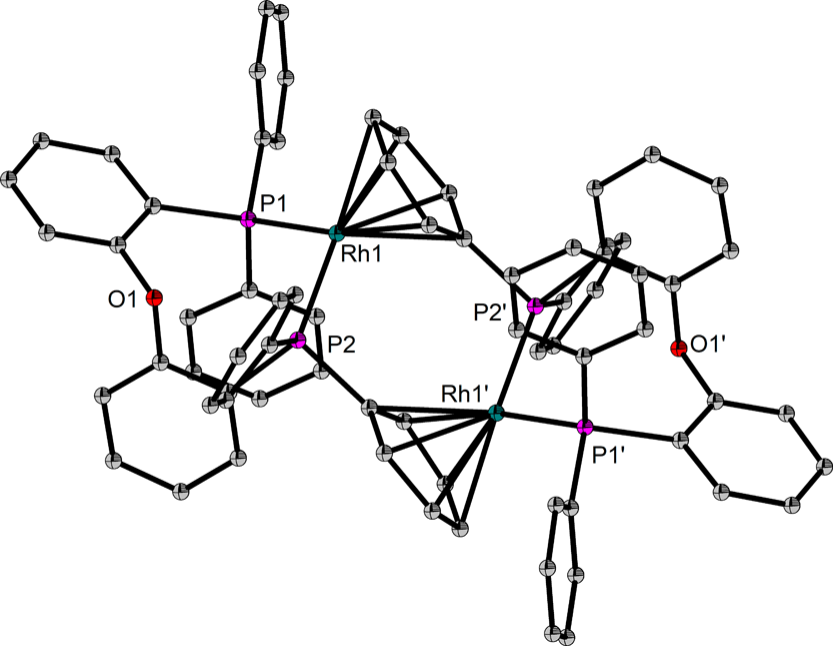
Molecular structure of the cationic part of [Rh(DPEPhos)]_2_[BF_4_]_2_ (**6**). Hydrogen atoms have been omitted for clarity (ORTEP, 50% probability ellipsoids). Selected bond length and angles: Rh–Rh 4.5958(5), Rh–P 2.2605(4)-2.2789(4) Å, Rh–C_Phenyl_ 2.2764(14)-2.3924(14) Å, P–Rh–P 95.314(15)°.

Interestingly the catalytic BTAH-allene coupling reaction can not only be carried out with the literature-described parent neutral system [Rh(μ-Cl)(DPEPhos)]_2_**1**, but also using the cationic arene-bridged complex [Rh(DPEPhos)]_2_[BF_4_]_2_ (**6**, Figure S24). This complex was identified, analyzed, and crystallized from CH_2_Cl_2_ for the first time in this work (Scheme 3, Figure 8) and represents an example of an arene-bridged dimer.

Formation of such complexes is commonly observed for arene-appended Rh diphosphine systems in the absence of stabilizing ligands.^32−34^ On a general note, arene complexes are known for a variety of transition metals and represent an interesting current field of research with relevance as deactivation products or catalyst precursors.^35−37^ Complex **6** is the first structurally characterized example of an arene bridged dimer of the type [Rh(PP)]_2_[BF_4_]_2_ in which the Rh-PP unit is not a five-membered chelate. Compared to neutral precatalyst **1**, a reduced activity has been observed for **6** (Figure S24). The ^31^P NMR spectra reveal that more inactive species **5** has been formed in the latter case (Figure S25). However, the fact that the catalytic BTAH-allene coupling reaction works with both neutral and cationic complexes suggests that Cl has only limited influence on the catalytic reaction and the formation of cationic intermediates during catalysis but could stabilize the catalytically relevant species by preventing the formation of homocoupling product **5**.

**Figure 9:**
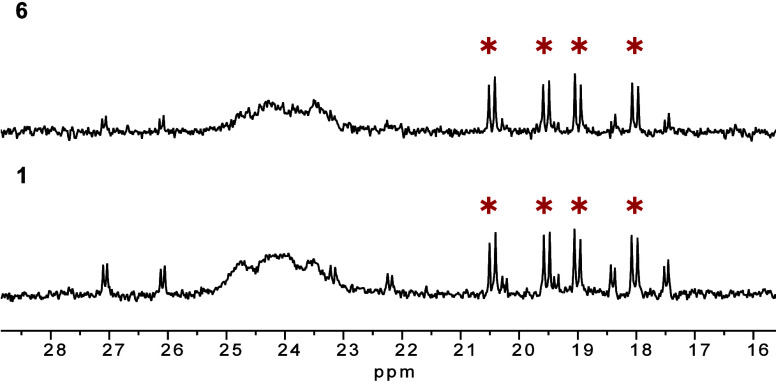
^31^P{^1^H} NMR spectra of (a) the neutral precatalyst [Rh(μ-Cl)(DPEPhos)]_2_**1** (0.01 mol·L^–1^) and (b) the cationic precatalyst [Rh(DPEPhos)]_2_[BF_4_]_2_**6** (0.01 mol·L^–1^), each converted with an excess of BTAH (0.3 mol·L^–-1^) and cyclohexyl allene (0.4 mol·L^–1^). Signals labeled with ∗ correspond to the catalytically inactive species **5**.

The reaction of both neutral and cationic precatalysts with both substrates BTAH and cyclohexyl allene produces virtually the same ^31^P{^1^H} NMR spectra at room temperature (Figure 9) and at 50 °C (Figure S25). The most intense signal in both spectra is found between 23 and 25 ppm. Besides this unknown species, the Rh(III)-π-allyl-vinyl-complex **5** is present as well as signals at δ 18.4 and 26.8 ppm that correspond to formation of allene complexes that were already observed by adding an excess of allene to the precatalyst (without BTAH addition, vide supra). Furthermore, as detailed above, heterolytic Rh–Cl bond cleavage and Cl^–^ dissociation was observed in control reactions of the neutral precatalyst with cyclohexyl allene and BTAH. We therefore exclude a mechanistic scenario based exclusively on neutral Rh complexes as shown in Figure 1b.

#### Investigation of Proton Transfer

BTAH coordination instead of initially proposed oxidative addition^13^ as a key step of a potential catalytic cycle is only reasonable if the BTAH N–H proton is transferred to the allene through a direct protonation sequence, avoiding terminal Rh hydride intermediates at early stages of catalysis.^38−44^ For direct proton transfer to occur, both substrates (BTAH and allene) must coordinate to the Rh center, and the *N*^1^-BTAH must undergo isomerization to form neutral [Rh(Cl)(DPEPhos)(*N*^2^-BTAH)(1,2-allene)] or cationic [Rh(DPEPhos)(*N*^2^-BTAH)(1,2-allene)]Cl.

To elucidate this reaction sequence experimentally, we performed the catalytic BTAH–allene coupling reaction under otherwise identical conditions in the presence of an additional proton source. Such control experiments were successfully used to support catalytic transformations involving proton shuttling events.^38^ Pyridinium *p*-toluenesulfonate (PPTS) was chosen for this purpose since it is known to support the rhodium complex-catalyzed coupling of benzotriazoles to terminal alkynes.^45,46^ Before the catalytic reaction was performed with PPTS as an additive, the substrate free reaction of the acid and the precatalyst **1** was investigated. According to ^31^P{^1^H} NMR analysis (Figure S26), several species were formed of which one could be assigned to the bis(pyridine) species [Rh(DPEPhos)(py)_2_]Cl (py = pyridine; d, 37.5 ppm; *J*_RhP_ = 176 Hz), which could also be isolated and crystallographically characterized (Figure S27). This species corresponds to the first rhodium pyridine solvent complex of the type [Rh(diphosphine)(py)_2_]^+^. Recently, Chirik *et al*. described the formation and isolation of a similar Co(I) complex [Co(^*i*^Pr-DuPhos)(py)_2_][BAr^F^_4_].^47^

NMR spectroscopic monitoring of a proton supported catalytic coupling reaction shows that this transformation already occurs at room temperature within approximately 12 h (Figure S28), in significantly milder conditions than reported before.^1^ Interestingly, the ^31^P{^1^H} NMR spectrum at room temperature (Figure S29) reveals the same dd, in this case more intense, that was found at −25 °C for the PPTS-free reaction mixture. Assuming this to be a catalytically active species, the additional protons seem to have a stabilizing effect on the same. The reaction progress of the PPTS supported catalytic reaction was monitored at room temperature by using low-field NMR spectroscopy (Figure S30). In contrast to the original procedure, *no* sigmoidal behavior of substrate consumption/product formation was observed. Instead, both processes appear to follow a simple zero-order scenario (Figure S31).^48^ To further support the beneficial role of the proton source for catalysis, addition of a proton scavenger such as Hünig’s base (*N,N*-diisopropylethylamine) fully inhibits catalysis (Figure S32). The precatalyst itself does not react with the base, as verified in a separate control experiment (Figure S33).

#### Computational Investigations

The unexpected experimental results for all stages of the catalytic reaction motivated the design of an all-encompassing DFT study that initially addressed the following questions: (i) What is the first step of the mechanistic scenario? (ii) Are cationic species formed during catalysis, and what is the influence of the chloride ion? (iii) How is the proton transferred from the BTAH to the allene moiety? (iv) What are the inhibiting species? (v) What is the deactivation mechanism, and how does chloride affect it? (vi) What does the complete reaction path look like?

We have chosen a multilevel quantum mechanical approach to answer the questions that have arisen during the experimental investigations as efficiently and accurately as possible.^49^ The preoptimizations and reaction path analysis were performed using xTB 6.5.1^50^ (GFN2-xTB),^51^ the obtained structures were reoptimized and checked at the DFT level with Gaussian16^52^ (B3LYP^53−58^-D3^59,60^/def2-SVPP/298 K),^61^ and single-point calculations were performed to account for solvent correction with the same basis set (SMD,^62^ 1,2-DCE) and to obtain more accurate electronic energies using ORCA^63−65^ (DLPNO–CCSD(T)^66−70^/def2-TZVP, final notation DLPNO–CCSD(T)/def2-TZVP/SMD//B3LYP-D3/def2-SVPP).

##### i The First Step: Substrate Coordination

**Figure 10:**
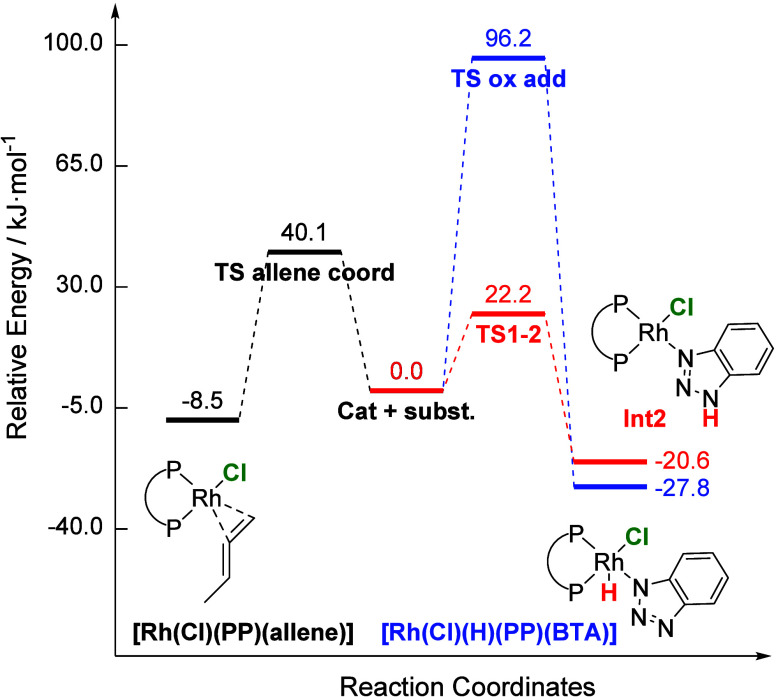
Calculation of possible reactions of [Rh(Cl)(DPEPhos)] with BTAH and Me-allene (DLPNO–CCSD(T)/def2-TZVP/SMD//B3LYP-D3/def2-SVPP).

In previous publications, it was assumed that catalysis is initiated by oxidative addition of BTAH to the monomerized precatalyst **1a**.^13^ In contrast, we experimentally confirmed that BTAH coordinates to the Rh center via the free electrons on the nitrogen. Similarly, we could show that cyclohexyl allene can coordinate to the catalyst. Thermodynamic considerations of all three possible sequences indicate that the reaction of BTAH with the monomerized precatalyst is, in both cases, energetically favored over allene coordination (Figure 10).

Oxidative addition and coordination of BTAH lead to energetically similar intermediates, of which the oxidative addition is thermodynamically slightly favored (ΔΔ_R_*G* = 7.2 kJ·mol^–1^). However, the latter proceeds via a significantly higher energy barrier (Δ*G*^‡^_TSoxadd_ = 96.2 kJ·mol^–1^), compared to that for BTAH coordination (Δ*G*^‡^_TS1–2_ = 22.2 kJ·mol^–1^). Thus, oxidative addition seems to be kinetically hindered compared with coordination, supporting the experimental findings. It can be concluded that coordination of BTAH to **1a** is likely to be the first step in the mechanism. Alternative coordination modes for BTAH and allene have been calculated and can be found in Figure S34.

##### ii Relevance of Cationic Species for Catalysis and Role of Chloride

The experimental study showed that when both substrates are present in excess, the catalyst **1a** forms cationic species while Cl^–^ acts as a counterion.

**Figure 11:**
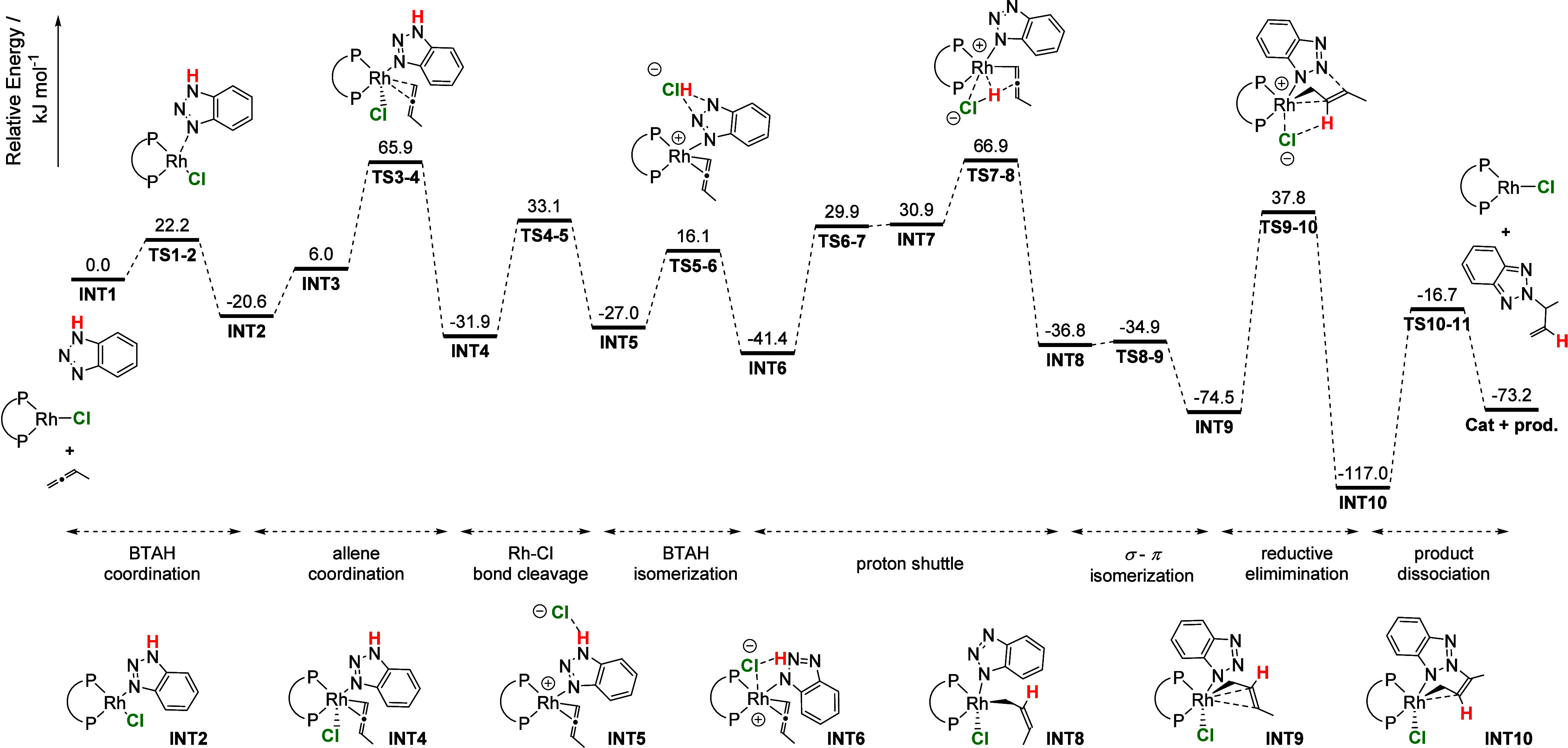
Gibbs free energy profile (Δ*G*/kJ·mol^–1^) for the coupling of BTAH and Me-allene starting from optimized SC-XRD structure of **2** (here **INT2**) (DLPNO–CCSD(T)/def2-TZVP/SMD//B3LYP-D3/def2-SVPP).

To investigate whether a similar reactivity is possible during the catalytic reaction, the heterolytic Rh–Cl bond cleavage at [Rh(Cl)(DPEPhos)(allene)(BTAH)] (**INT4**) was calculated and an activation barrier of 65.0 kJ·mol^–1^ was found (Figure 11). The resulting formal cationic complex [Rh(DPEPhos)(allene)(BTAH)]Cl (**INT5**) is stabilized by a chloride ion hydrogen-bonded to the BTAH proton.

A similar stabilizing effect of an N–H hydrogen bond to a Cl^–^ counterion was recently described by Weller and co-workers for the diolefin complex [Rh(κ^3^-(^*i*^Pr_2_PCH_2_CH_2_)_2_NH)(NBD)]Cl.^71^ Comparing the cationic and neutral complex, only a very small energy difference was calculated (ΔΔ_R_*G* = 4.9 kJ·mol^–1^). According to these calculations, cationic species are energetically accessible and do not lead to a destabilization of the complex.

##### iii Proton Transfer from the BTAH to the Allene Moiety

**Figure 12:**
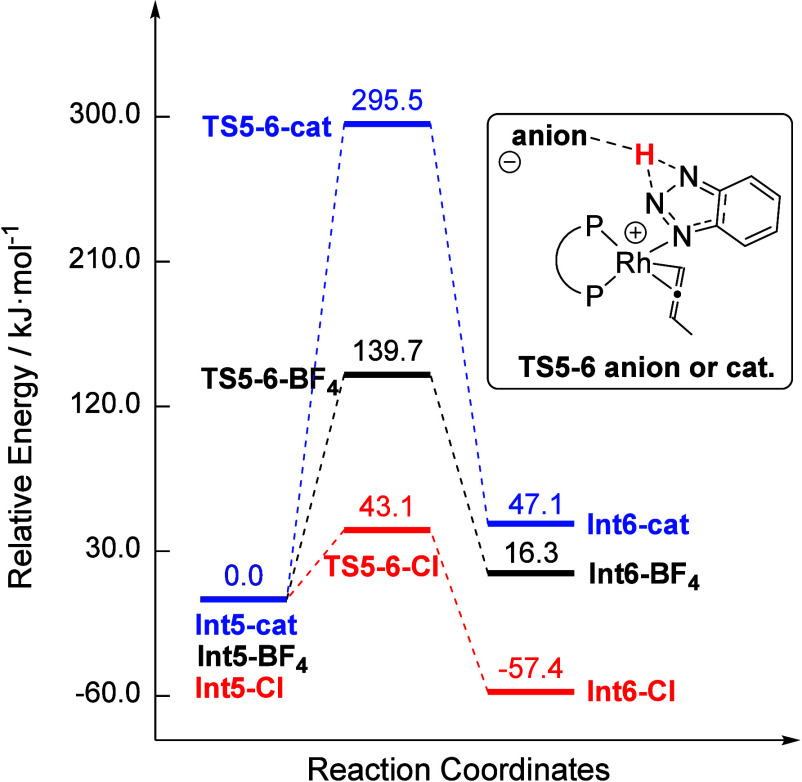
Isomerization of coordinated BTAH for cationic **INT5-cat** as well as for its [BF_4_]^−^ and Cl^–^ salts (DLPNO–CCSD(T)/def2-TZVP/SMD//B3LYP-D3/def2-SVPP).

If the oxidative addition of BTAH is excluded, alternative proton transfer mechanisms must be considered. Control experiments suggested a protonation of the coordinated allene. Using the geometry of the experimental SC-XRD structure **2**, a reaction path of a possible isomerization/proton shuttling event was calculated. To realize the experimentally found *N*^2^ selectivity, it is essential that the proton in BTAH is located on the *N*^2^ while the molecule is *N*^1^ coordinated to Rh. The corresponding isomerization was calculated for the cationic complex [Rh(DPEPhos)(allene)(BTAH)]^+^, leading to an energetically inaccessible transition state of Δ*G*^‡^_TS5–6-cat_ = 295.5 kJ·mol^–1^ (Figure 12). The resulting *N*^2^-isomer of the complex **INT6-cat** is moreover disfavored due to a dearomatization of the BTAH. Interestingly, the energy of this transition state can be drastically reduced by stabilization with counterions such as [BF_4_]^−^ and especially Cl^–^.^44^ For the latter, a transition state of only Δ*G*^‡^_TS5–6-Cl_ = 43.1 kJ·mol^–1^ was found together with an exergonic reaction toward the *N*^2^-isomer **Int6-Cl** (Δ_R_*G*_INT6-Cl_ = −57.4 kJ·mol^–1^).

This finding underlines the importance of Cl decoordination during catalysis and the formation of cationic species. Furthermore, the higher-lying transition state in the case of the [BF_4_]^−^ counterion could be one reason for the lower catalytic activity of **6** compared to **1**. Similar calculations were performed for the free BTAH molecule, showing the same trend. Here, the influence of the tosyl anion (from PPTS) on the isomerization was investigated, leading to a comparable type of stabilization of the transition state (Figure S35). After isomerization, the protonation step takes place. From **INT6**, it was found that Cl^–^ as a counterion once more supports the H shift and is essential for the proton shuttling event (**TS7–8,**Figure 11). According to the calculations, the protonation proceeds via a barrier-free formation of **INT7** (Δ_R_*G*_INT6→INT7_ = 72.3 kJ·mol^–1^) in which the H–Cl unit is stabilized on the Rh center. A second transition state (Δ*G*_INT7→TS7–8_ = 36.0 kJ·mol^–1^) was found as well as **INT8**, a σ-allyl complex. In general, this type of ligand-assisted proton shuttling (LAPS)^72^ as a special case of metal–ligand cooperation has been proposed before for various catalytic and stoichiometric transformations.^73−75^ Examples for Rh(I) catalysis are however scarce.^39^

##### iv Inhibiting Species

**Scheme 4:**
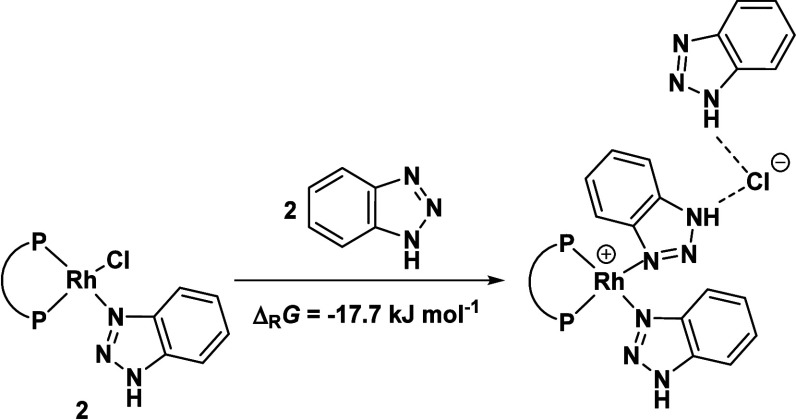
Hydrogen Bonding of BTAH and Chloride, Leading to BTAH Inhibition of Catalysis (DLPNO–CCSD(T)/def2-TZVP/SMD//B3LYP-D3/def2-SVPP)

It was found experimentally that an increase in BTAH concentration leads to a reduced catalytic activity, and it was proposed that the formation of the cationic di-BTAH complex leads to the inhibition of catalysis. Consequently, the BTAH coordination event to **2** and the concomitant heterolytic Rh–Cl bond cleavage was investigated computationally. Different possible descriptions of the same reaction were calculated (Figure S36). It was found that the formation of the cationic species is favored when the Cl^–^ is stabilized by hydrogen bonding to the NH unit of BTAH. With an excess of BTAH, it is possible that the counterion is stabilized by coordinated and additional free BTAH (Δ_R_*G* = −17.7 kJ·mol^–1^, Scheme 4). The influence of free BTAH on the stabilization of **3-Cl** could explain the inhibition observed in the *in operando* experiments.

##### v Deactivation Mechanism and Role of Chloride

**Scheme 5:**
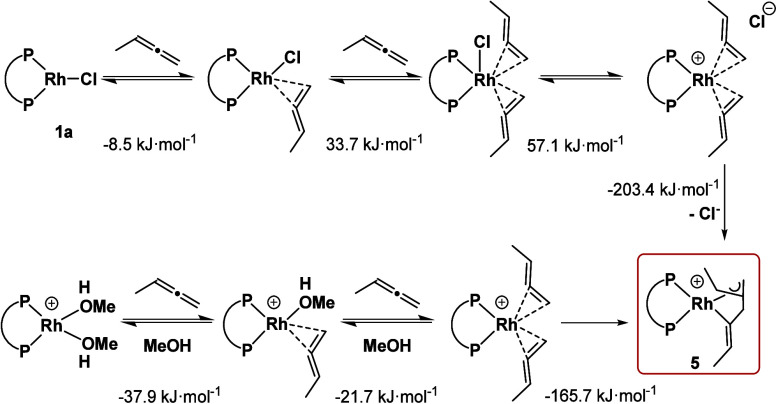
Stepwise Formation of Cation **5** from **1a** or [Rh(DPEPhos)(MeOH)_2_]^+^ and the Calculated Δ_R_*G* for All Reaction Steps (DLNPO–CCSD(T)/def2-TZVP/SMD// B3LYP-D3/def2-SVPP)

The reaction of the precatalyst **1** with an excess of allene was found to lead to the formation of an inactive 18-electron species, π-allyl-σ-vinyl-complex **5**. It was found that this species can also be formed by the stoichiometric addition of allene to [Rh(DPEPhos)(Solv)_2_]^+^. Both reaction paths were calculated by using methyl allene as a model substrate (Scheme 5). While in the cationic case all reaction steps are exergonic, the neutral pathway shows endergonic intermediates. This might cause stronger deactivation and slower catalysis when **6** is used as a catalyst instead of **1**. In both cases, the coupling event that forms rhodacycle **5** is the most energetically favorable step.

The TS of that coupling event was calculated and found to be accessible at room temperature (Figure S37, Δ*G*^‡^ = 64.7 kJ·mol^–1^). Comparing the reaction of the model substrate Me-allene to the allenes used in the experimental study (*n*-pentyl allene and cyclohexyl allene), no significant energy differences could be found (Figure S37).

##### vi Proposed Reaction Mechanism

**Figure 13:**
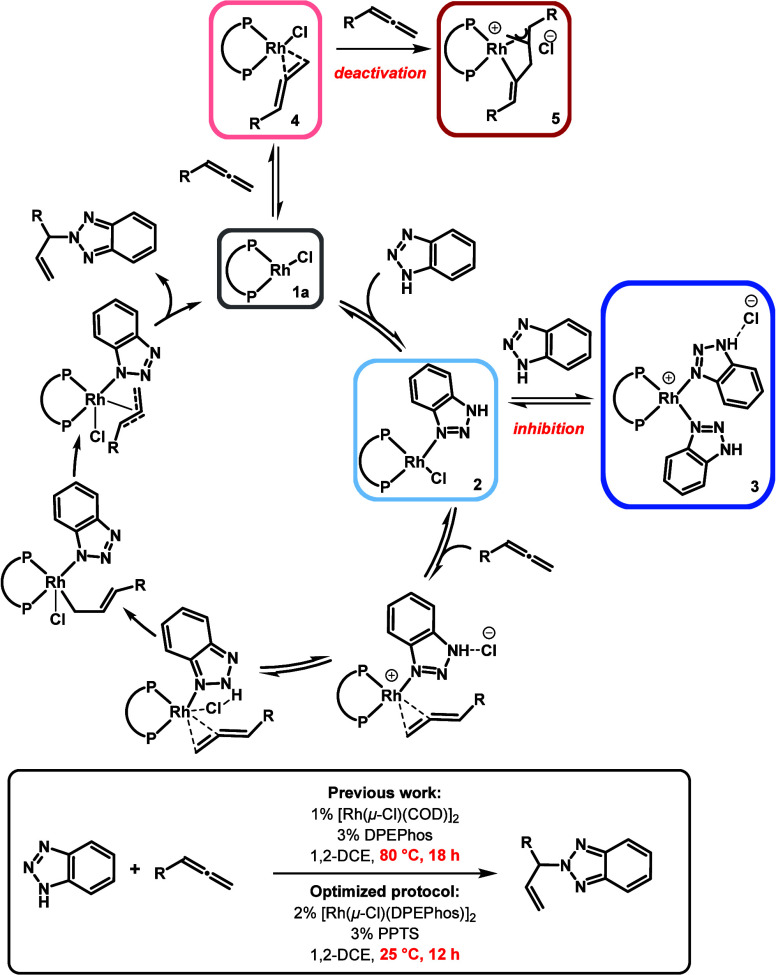
Top: Proposed revised catalytic cycle of the Rh complex catalyzed coupling of BTAH to allenes. Bottom: Optimized reaction conditions.

Taken together, the combined experimental and computational results of this study lead to a new understanding of the mechanistic pathway (Figures 11 and 13). We propose that in the first step, the assumed monomerized precatalyst **1a**(76) coordinates BTAH via the *N*^3^-lone pair, producing a Rh(I)-monobenzotriazole complex **2**/**INT2**. Allene is coordinated in the second step, giving **INT4**. For both coordination steps, low-lying transition states have been found (Δ*G*^‡^_TS1–2_ = 22.2 kJ·mol^–1^, Δ*G*^‡^_TS3–4_ = 65.9 kJ·mol^–1^). The Rh–Cl bond is cleaved, and the cationic substrate complex **INT5** is formed (Δ*G*_INT5_ = −27.0 kJ·mol^–1^). The decoordination of Cl^–^ is essential for the following steps of proton shift, including first an isomerization of the coordinated BTAH (**INT6**) followed by a proton shuttling step from the BTAH to the coordinated allene. **INT8**, a σ-allyl complex, isomerizes and forms a π-allyl complex in an almost barrier free step. The latter is lower in energy (ΔΔ_R_*G* = 37.7 kJ·mol^–1^). Reductive elimination of the *N*^2^-product (Δ*G*^‡^_TS9–10_ = 37.8 kJ·mol^–1^) results in formation of a product complex **INT10** (Δ_R_*G*_INT10_ = −117.0 kJ·mol^–1^).

Product release regenerates catalytically active species **1**. A summary of the revised catalytic cycle for BTAH coupling to allenes at [Rh(DPEPhos)]^+^ with the key steps of BTAH coordination, allene coordination, BTAH isomerization, proton shuttling, and reductive elimination is shown in Figure 13. Based on the mechanistic studies that led to this proposed scenario, the reaction conditions for the BTAH-allene coupling could be optimized. The reaction temperature could be drastically reduced from 80 °C to room temperature in the presence of PPTS as an external proton source. Furthermore, reactions are much faster (12 h instead of 18 h) and substrate inhibition can be fully eliminated.

## Conclusions

In summary, we have presented a detailed experimental and computational study of the mechanism of [Rh(DPEPhos)]-catalyzed coupling of benzotriazole and allenes. Our data suggest a mechanistic scenario in which a rare case of counterion-assisted isomerization of the heteroarene and proton shuttling between both substrates are the key steps instead of previously suggested oxidative addition of benzotriazole. Our experimental data support an allene-based deactivation pathway, leading to formation of a novel rhodacyclic species as well as substrate inhibition by benzotriazole. The identification of proton shuttling as a key step led to the introduction of PPTS as an external proton source into the catalytic process. As a consequence, the reaction temperature could be drastically reduced from 80 °C to room temperature, and full conversion could be observed in much shorter reaction times. Although not verified experimentally in this study, another option to reduce the reaction temperature could be to add the substrates in a stepwise fashion or even continuously, *e.g*., using a syringe pump. In doing so, allene-based deactivation and BTAH-based substrate inhibition can be minimized, and the reaction can be performed at room temperature.

The presented results impressively show how neutral and cationic species can coexist and even be in equilibrium with each other during catalysis. This fact generally complicates the mostly empirical design of catalytic cycles, where such effects have to be taken into account.

## Supporting Information Available

Experimental, spectroscopic, crystallographic, and computational details (PDF)
Calculated geometries (XYZ)

## Supplementary Material

ja4c02679_si_001.pdf

ja4c02679_si_002.xyz

## ABBREVIATIONS

BTAH: benzotriazole
Cy: cyclohexyl
1,2-DCE: 1,2-dichloroethane
DPEPhos: bis[(2-diphenylphosphino)phenyl]ether
JoSPOPhos: (1*R*)-1-[(*R*)-(1,1-dimethylethyl)phos-phinyl]-2-[(1*R*)-1-(diphenylphosphino)-ethyl]ferrocene
PPTS: pyridinium *p*-toluenesulfonate
SMD: solvent model based on density
