# Supplementary material for: The Mechanism of Rh(I)-Catalyzed Coupling of Benzotriazoles and Allenes Revisited: Substrate Inhibition, Proton Shuttling, and the Role of Cationic vs Neutral Species
Source: J Am Chem Soc. 2024 Apr 22;146(17):12185–96. doi: 10.1021/jacs.4c02679 (PMC11066875; doi:10.1021/jacs.4c02679)
Supplement: Supplementary file 1 — ja4c02679_si_001.pdf [file ja4c02679_si_001.pdf]

Supporting Information

The Mechanism of Rh(I) Catalyzed Coupling of Benzotriazoles and Allenes Revisited: Substrate Inhibition, Proton Shuttling and the Role of Cationic vs. Neutral Species

Nora Jannsen*, Fabian Reiß, Hans-Joachim Drexler, Katharina Konieczny, Torsten Beweries*, Detlef Heller*

Leibniz-Institut für Katalyse e.V., Albert-Einstein-Str. 29a, 18059 Rostock, Germany

Table of contents

1 General information 2

2 Synthetic procedures 2

3 Supplementary NMR spectra and figures 6

4 Crystallographic data 23

5 DFT calculations 27

6 References 42

1 General information

All steps were carried out under oxygen- and moisture-free conditions under argon using standard Schlenk techniques. THF(-d_8), benzene(-d_6), diethyl ether and *n*-heptane were distilled from sodium and 1,2-DCE(-d_4) from CaH_2. The subsequent removal of oxygen traces was carried out by applying six freeze-thaw cycles. The rhodium precursor [Rh(COD)(μ_2-Cl)]$_2$ (STREM, 98 %), used in this study was recrystallized from CH_2Cl_2. The diphosphine ligand DPEPhos (bis[(2-diphenylphosphino)phenyl]ether) (TCI, 98 %) was used without further purification. ^{1}H NMR and ^{31}P NMR spectra were obtained on a Bruker AV300 or AV400 spectrometer at 297-298 K (unless otherwise specified) and were referenced internally to the deuterated solvent.

2 Synthetic procedures

This section reports the spectroscopic details of the complexes used for this study. The Rh complexes were either reported before (references are given) or represent catalytically relevant intermediates that were difficult to isolate and purify, *e.g.*, due to incomplete conversion or to co-crystallization of starting material or other Rh complexes.

2.1 [Rh(DPEPhos)(COD)]BF$_4$

[Rh(DPEPhos)(COD)]BF$_4$ was synthesized according to a literature known procedure.[1] [Rh(ACAC)(COD)] (31.2 mg, 0.1 mmol) and DPEPhos (53.8 mg, 0.1 mmol) were separately dissolved in THF (5 mL, each). The complex solution was cooled to -78 °C and the ligand was added dropwise. The reaction solution was stirred at -78 °C for 1 h. Afterwards, a solution of HBF_4 in Et_2O (8 M, 125 µL) was added at -78 °C. The reaction mixture was stirred for another hour and then warmed to room temperature. The solution was layered with Et_2O, and [Rh(DPEPhos)(COD)]BF$_4$ crystallized. The red crystals were washed twice with Et_2O.

^{1}H NMR (300 MHz, MeOH-d_4): 8.00–6.74 (28H, m), 7.15–6.94 (1H, m), 4.59–4.38 (4H, m), 2.42–2.01 ppm (8H, m).

^{31}P NMR (122 MHz, MeOH-d_4): 13.2 ppm (d, J_{PRh} = 147 Hz).

2.2 [Rh(DPEPhos)(MeOH)$_2$]BF$_4$

[Rh(DPEPhos)(COD)]BF$_4$ (8.6 mg, 0.01 mmol) was dissolved in 1 mL of MeOH and hydrogenated for 30 s (due to its pre-hydrogenation time).[2, 3] Hydrogen was removed from the red solution in three freeze-pump-thaw cycles (NMR yield >99%).

^{1}H NMR (300 MHz, in MeOH-d_4): 7.66-7.57 (6H, m); 7.49-7.23 (16H, m); 6.99-6.91 (2H, m), 6.82 (2H, t, 7.6 Hz), 6.63-6.54 ppm (2H, m).

^{31}P NMR (122 MHz, in MeOH-d_4): 44.9 ppm (d, J_{PRh} = 213 Hz).

2.3 [Rh(μ-Cl)(DPEPhos)]$_2$ (1)

[Rh(μ-Cl)(DPEPhos)]$_2$ was synthesized according to a literature known procedure.[4] [Rh(μ-Cl)(COD)]$_2$ (55.7 mg, 0.113 mmol) and DPEPhos (121.7 mg, 0.226 mmol) were dissolved in 3 mL of 1,2-DCE and stirred for 30 min at room temperature. The solution was layered with Et_2O, leading to an amorphous crystallization. The red powder ([Rh(μ-Cl)(DPEPhos)]$_2$) was washed twice with *n*-heptane and dried in vacuum.

^{31}P NMR (121 MHz, in THF-d_8): ≈ 38 (d, J_{PRh} ≈ 200 Hz) ppm.

2.4 [Rh(Cl)(DPEPhos)(BTAH)] (2)

To synthesize [Rh(Cl)(DPEPhos)(BTAH)], [Rh(μ-Cl)(DPEPhos)]$_2$ (12.0 mg, 0.00864 mmol) and 45 µL of a 0.4 M BTAH solution in 1 mL of 1,2-DCE were stirred for 1 hour. An amorphous yellow solid was received overnight and washed twice with ca. 2 mL of Et_2O. The powder was dissolved in 0.5 mL of THF and 1 mL of 1,2-DCE and layered with 1 mL of *n*-heptane. Orange crystals, suitable for X-ray single crystal analysis were formed overnight.

^{31}P NMR (122 MHz, in THF-d_8/1,2-DCE-d_4, 222 K): 38.5 (dd, J_{PRh} = 171 Hz, J_{PP} = 48 Hz); 35.3 (dd, J_{PRh} = 202 Hz, J_{PP} = 48 Hz) ppm (NMR spectra show additional signals of **1** and **3**).

*Note: No further analytical data are given as complex **2** cannot be isolated in pure form due to the presence of complexes **1** and **3**.*

2.5 [Rh(DPEPhos)(BTAH)$_2$]BF_4 (3)

[Rh(DPEPhos)(COD)]BF_4 (8.4 mg, 0.01 mmol) was dissolved in 2 mL of MeOH and hydrogenated for 33 seconds due to its pre-hydrogenation time. Hydrogen was removed in three freeze-pump-thaw cycles. Benzotriazole (2.7 mg, 0.023 mmol) was added to the solution and the mixture was stirred for one hour. The solvent was removed in vacuum and the residue was dissolved in 0.6 mL of THF and layered with 5 mL of Et_2O. After a couple of days, orange crystals could be isolated and analyzed by X-ray single crystal analysis.

^{1}H NMR (300 MHz, in THF-d_8): 8.03 (2H, d, J = 8.2 Hz); 7.88 (18H, dd, J = 3.2 Hz, J = 6.2 Hz, BTAH); 7.49 (20H, dd, J = 3.0 Hz, J = 6.3 Hz, BTAH); 7.34-7.27 (3H, m); 7.27-7.16 (7H, m); 7.09 (9H, broad d, J = 23.6 Hz); 7.09 (3H, broad d, J = 8.2 Hz); 6.84 (2H, t, J = 7.5 Hz); 6.76 (2H, dtd; J = 1.7 Hz, J = 4.3 Hz, J = 7.9 Hz) ppm.

^{31}P NMR (122 MHz, in THF-d_8): 36.2 (d, J_{PRh} = 179 Hz) ppm.

2.6 [Rh(π-allyl-σ-vinyl-cyclohexyl allene)(DPEPhos)]BF_4 (5)

[Rh(DPEPhos)(COD)]BF_4 (83.6 mg, 0.10 mmol) was dissolved in MeOH (10 mL) and the orange suspension was hydrogenated for 4 min. The hydrogen was removed from the dark red solution in three freeze-pump-thaw cycles. Cyclohexyl allene (29 µL, 0.2 mmol) was added and the solution was stirred for 90 min and additionally dried in vacuum. The residue was dissolved in 2.5 mL of MeOH and stored at 4 °C. Overnight an off-white precipitate formed, that was separated from the orange solution. The product was washed three times with Et_2O (ca. 5 mL) and finally dried in vacuum. Circa 43 mg (0.05 mmol) of product was isolated (49% yield). Colourless needles suitable for X-ray crystallography were grown from a saturated solution in MeOH.

^{1}H NMR (400 MHz, in MeOH-d_4): 7.94-7.16 (20H, m, Ph); 7.02 (4H, m, Ph); 6.46 (4H, m, Ph); 4.98 (1H, m, vinyl); 4.80 (1H, s, allyl); 4.07 (1H, m); 3.63 (1H, d, J = 15.7 Hz, -CH_2); 3.43 (1H, d, J = 15.6 Hz, -CH_2); 2.33 (1H, m, cyclohexyl); 2.18 (1H, m, allyl); 2.01-0.56 (20H, m, cyclohexyl) ppm.

^{31}P NMR (162 MHz, in MeOH-d_4): 20.5 (dd, J_{PRh} = 150 Hz, J_{PP} = 17 Hz); 19.0 ppm (dd, J_{PRh} = 160 Hz, J_{PP} = 17 Hz).

^{103}Rh NMR (13 MHz, in MeOH-d_4): 918 ppm.

HR-MS (ESI-TOF): calculated for $[C_{54}H_{56}OP_2Rh]^+$ m/z = 885.2856; found m/z = 855.2833.

2.7 $[Rh(DPEPhos)]_2(BF_4)_2$ (6)

[Rh(DPEPhos)(COD)]BF_4 (16. 8 mg, 0.02 mmol) was dissolved in 1.2 mL of DCM and hydrogenated for 2 min. Hydrogen was removed from the brown solution in three freeze-pump-thaw cycles. Brown crystals suitable for X-ray crystallography grew over night.

^{1}H NMR (400 MHz, in DCM-d_2): 8.14 (1H, t, J = 6.9 Hz); 8.04 (2H, m); 7.78-6.72 (46H, m); 6.34 (2H, m); 6.22 (1H, t, J = 7.3 Hz); 5.72 (1H, t, J = 7.3 Hz); 5.43 (1H, t, J = 6.8 Hz); 4.87 (1H, t, J = 7.8 Hz); 4.56 (1H, t, J = 7.2Hz) ppm.

^{31}P NMR (162 MHz, in DCM-d_2): 33.4 (dd, J_{RhP} = 124 Hz, J_{PP} = 22 Hz); 24.2 (dd, J_{RhP} = 176 Hz, J_{PP} = 41 Hz); 16.6 (dddd, J_{RhP} = 124 Hz, J_{PP} = 24 Hz, J_{PP} = 7 Hz); 7.14 (ddd, J_{RhP} = 181 Hz, J_{PP} = 41 Hz, J_{PP} = 29 Hz) ppm.

HR-MS (ESI-TOF): calculated for $[C_{72}H_{56}O_2P_4Rh_2]^{2+}$ m/z = 641.0665; found m/z = 641.0667.

2.9 High field monitoring of the complete catalytic reaction

[Rh(μ-Cl)(DPEPhos)]$_2$ (9.5 mg, 0.007 mmol) was dissolved in 1,2-DCE-d_4 (600 µL) and stirred for 30 minutes at room temperature. Cyclohexyl allene (60 µL, 0.4 mmol) was added to the precatalyst-benzotriazole mixture and the mixture was stirred for 30 seconds at room temperature. The solution

was transferred to a Young's tap NMR tube, in which BTAH (36.0 mg, 0.302 mmol) was placed. The measurements were performed at a reaction temperature of 50 °C.

2.10 Low field monitoring of the complete catalytic reaction

$[Rh(\mu\text{-}Cl)(DPEPhos)]_2$ (9.5 mg, 0.007 mmol) and BTAH (36.0 mg, 0.302 mmol) were dissolved in 1,2-DCE-d_4 (1200 µL) and stirred for 30 minutes at room temperature. Cyclohexyl allene (60 µL, 0.4 mmol) was added to the precatalyst-benzotriazole mixture and the mixture was stirred for 30 seconds at room temperature. The entire reaction mixture was transferred to a Young's tap NMR tube in which PPTS (2.8 mg, 0.01 mmol) was placed. The measurements were performed at room temperature.

3 Supplementary NMR spectra and figures

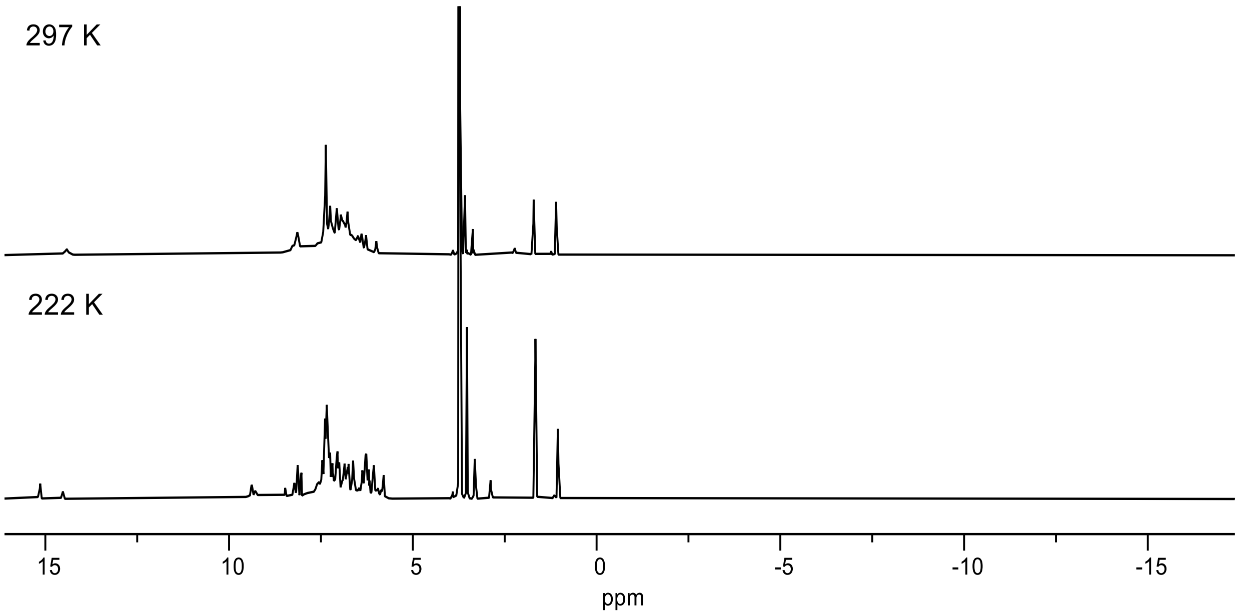

Figure S1. ^{1}H NMR spectrum (400 MHz, 297 K and 222 K) of dissolved crystals of the mono-BTAH complex [Rh(Cl)(DPEPhos)(BTAH)] (**2**) in 1,2-DCE-d_4/ THF-d_8.

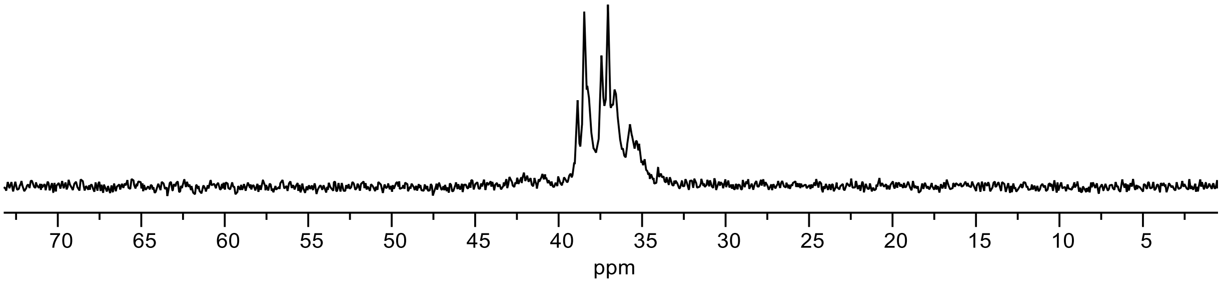

Figure S2. $^{31}P\{^{1}H\}$ NMR spectrum (122 MHz, 24 °C) of dissolved crystals of the mono-BTAH complex [Rh(Cl)(DPEPhos)(BTAH)] (**2**) in 1,2-DCE-d_4/THF-d_8.

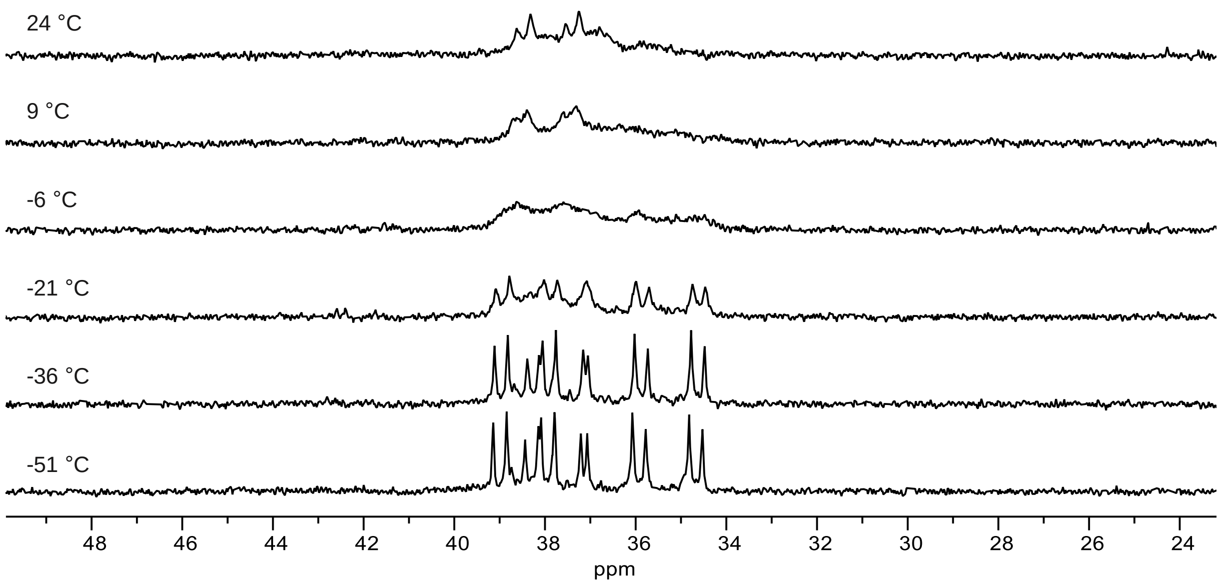

Figure S3. Variable temperature (162 MHz) $^{31}P\{^{1}H\}$ NMR spectrum of dissolved crystals of [Rh(Cl)(DPEPhos)(BTAH)] (**2**) in a mixture of THF-d_8 and 1,2-DCE-d_4.

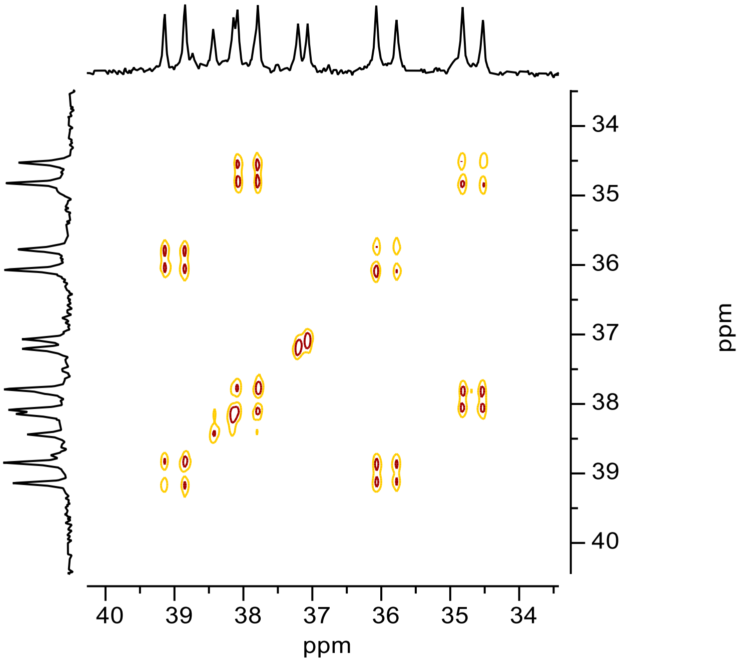

Figure S4. Low temperature (-51 °C) $^{31}P\{^{1}H\}$ COSY NMR spectrum (162 MHz) of dissolved crystals of the mono-BTAH complex [Rh(Cl)(DPEPhos)(BTAH)] (**2**) in 1,2-DCE-d_4/THF-d_8.

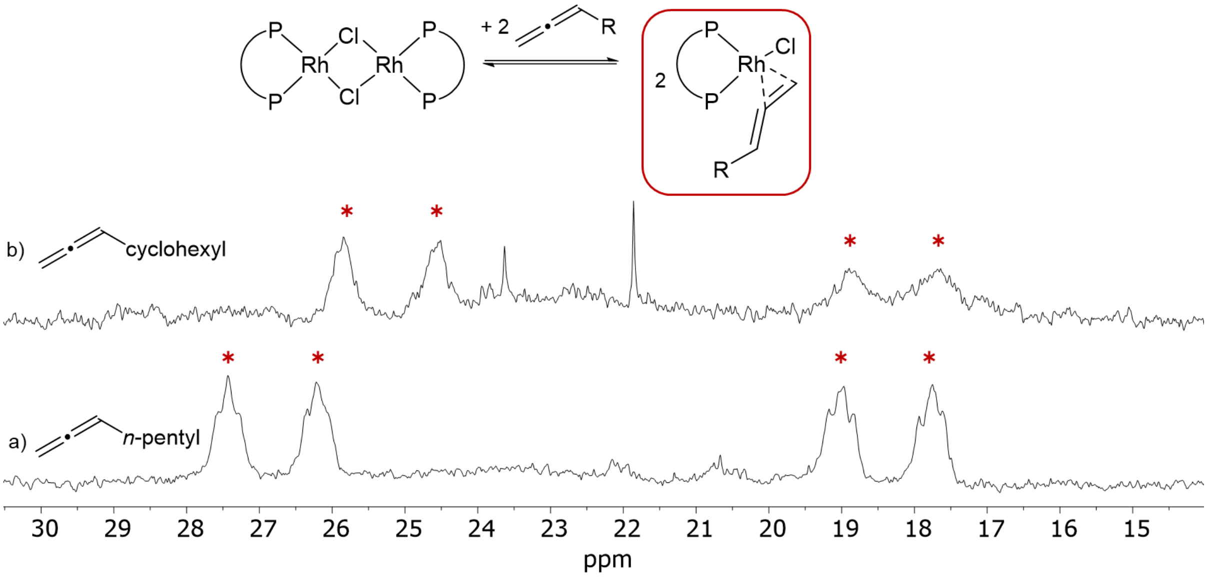

Figure S5. $^{31}P\{^{1}H\}$ NMR spectra of the conversion of **1** with 2 equiv. of a) *n*-pentyl allene and b) cyclohexyl allene.

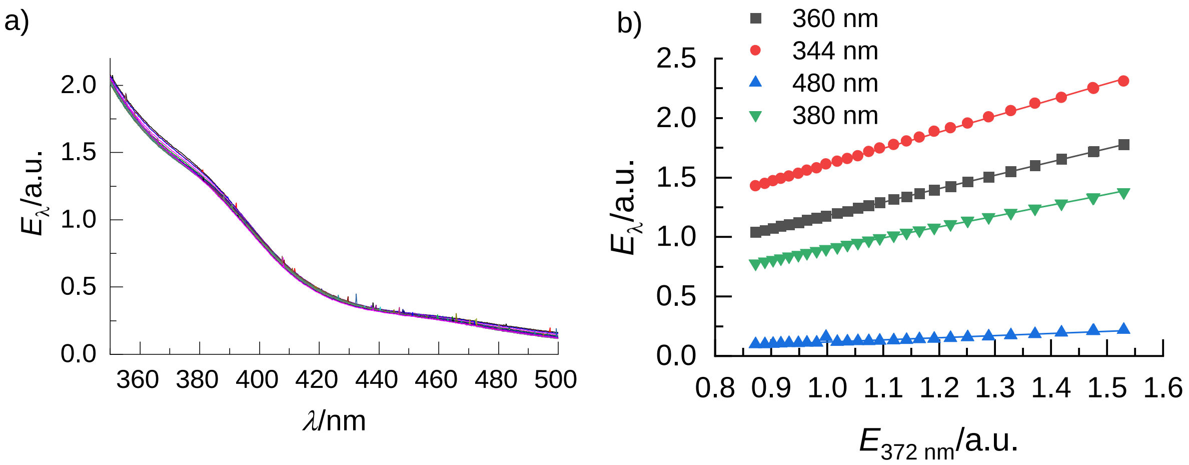

Figure S6. Titration of $5.32{\cdot}10^{-3}$ mmol **1** in 1,2 DCE with 2.5 mM *n*-pentyl allene solution (25.0 °C). a) Titration spectra, and b) extinction diagrams.

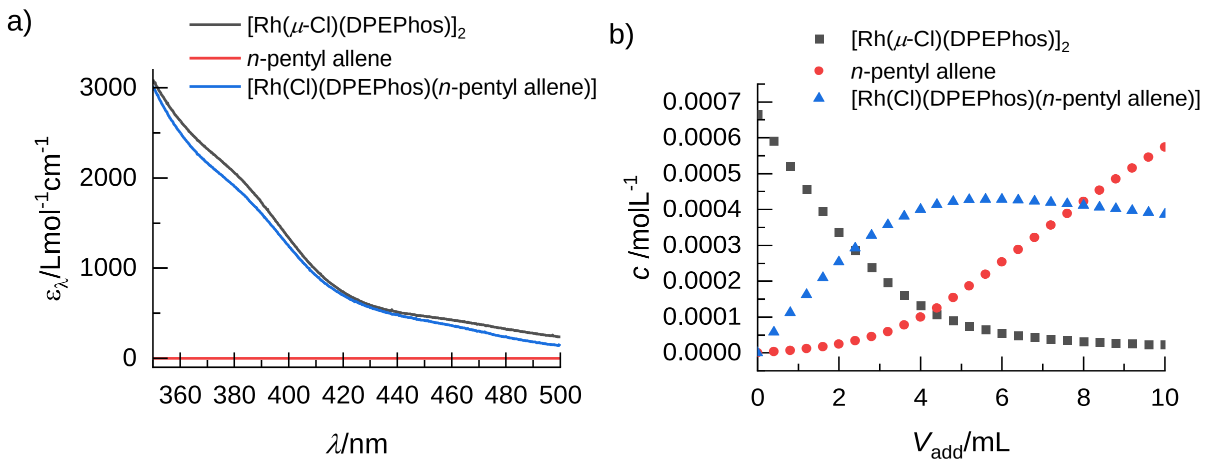

Figure S7. Evaluation of the UV-vis spectroscopic titration of **1** with *n*-pentyl allene in 1,2-DCE: a) pure component spectra, and b) concentration during the titration.

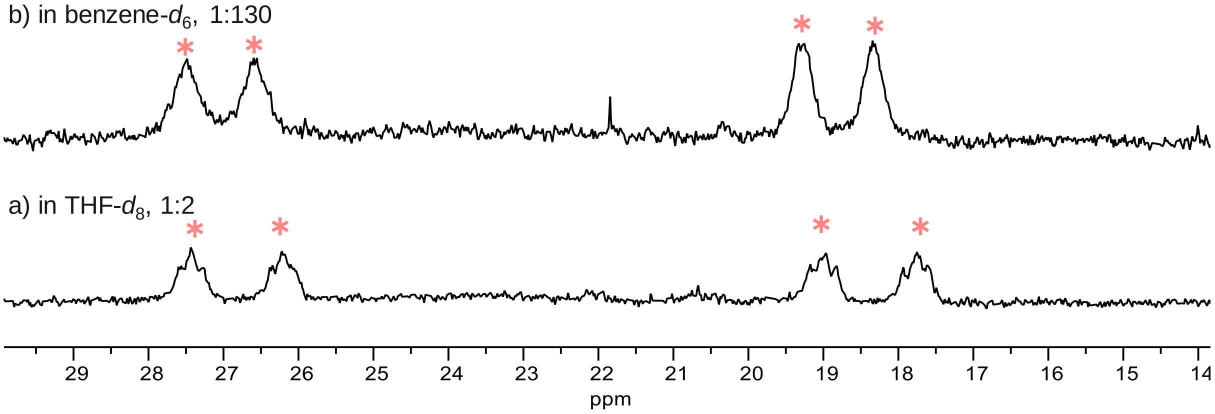

Figure S8. $^{31}P\{^1H\}$ NMR spectrum (122 MHz, 24 °C) of the conversion of [Rh(μ-Cl)(DPEPhos)]$_2$ **1** with 180 equivalents of *n*-pentyl allene in benzene-d_6.

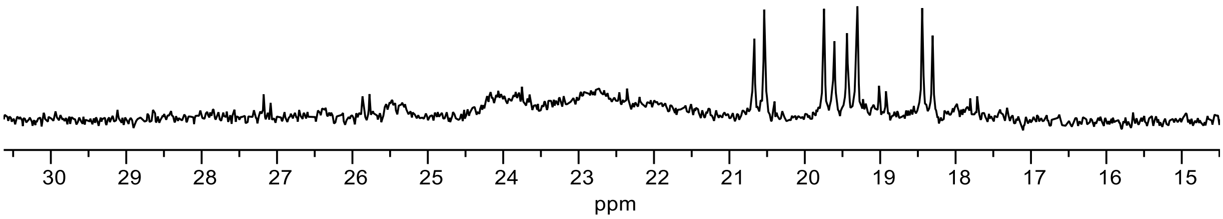

Figure S9. $^{31}P\{^1H\}$ NMR spectrum (122 MHz, 24 °C) of the conversion of **1** with 7 equivalents of cyclohexyl allene in THF-d_8.

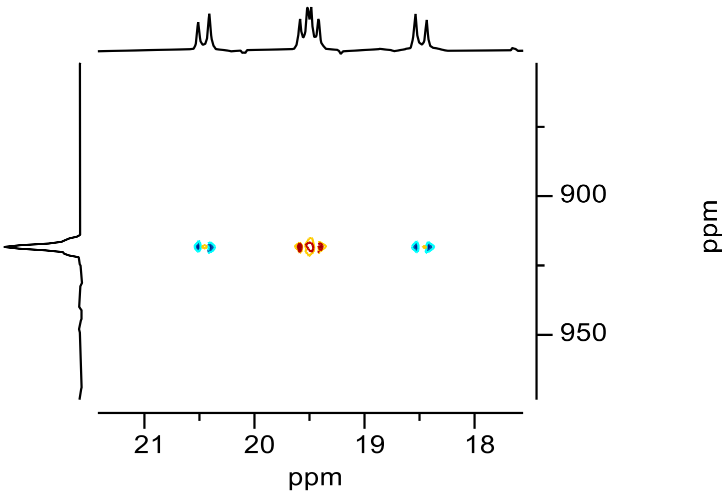

Figure S10. $^{31}P\{^1H\}$-$^{103}Rh\{^{31}P\}$-HMQC NMR spectrum (24 °C) of **5** (10 mmol·L^{-1}) in MeOH-d_4.

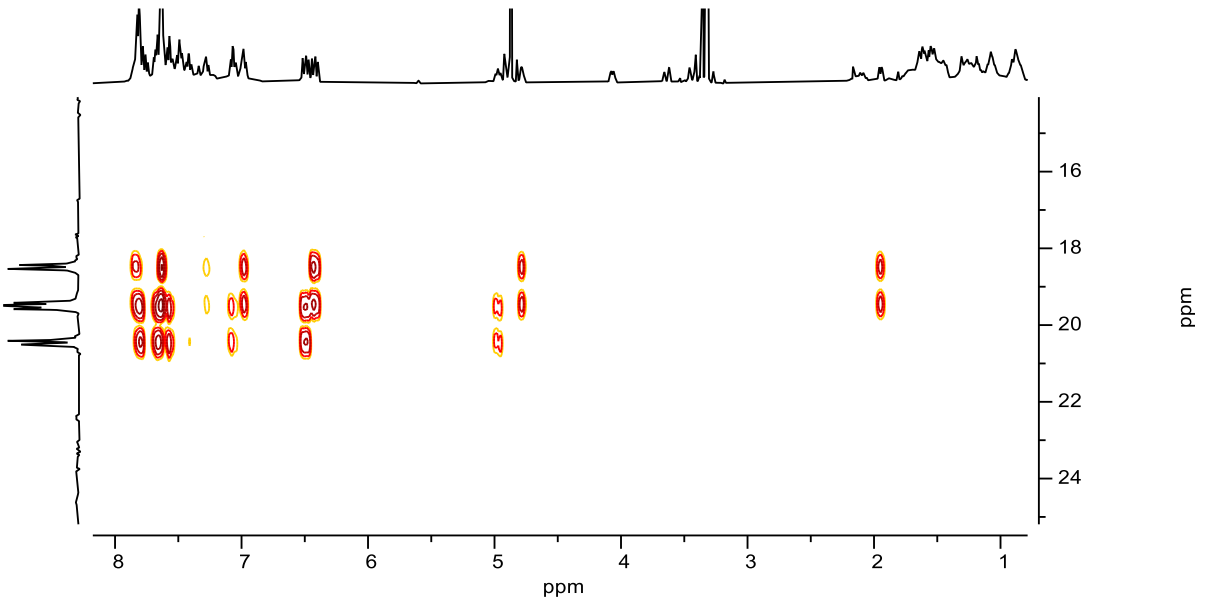

Figure S11. $^{1}H^{31}P\{^{1}H\}$ HMBC NMR spectrum (24 °C) of **5** (10 mmol·L^{-1}) in MeOH-d_4.

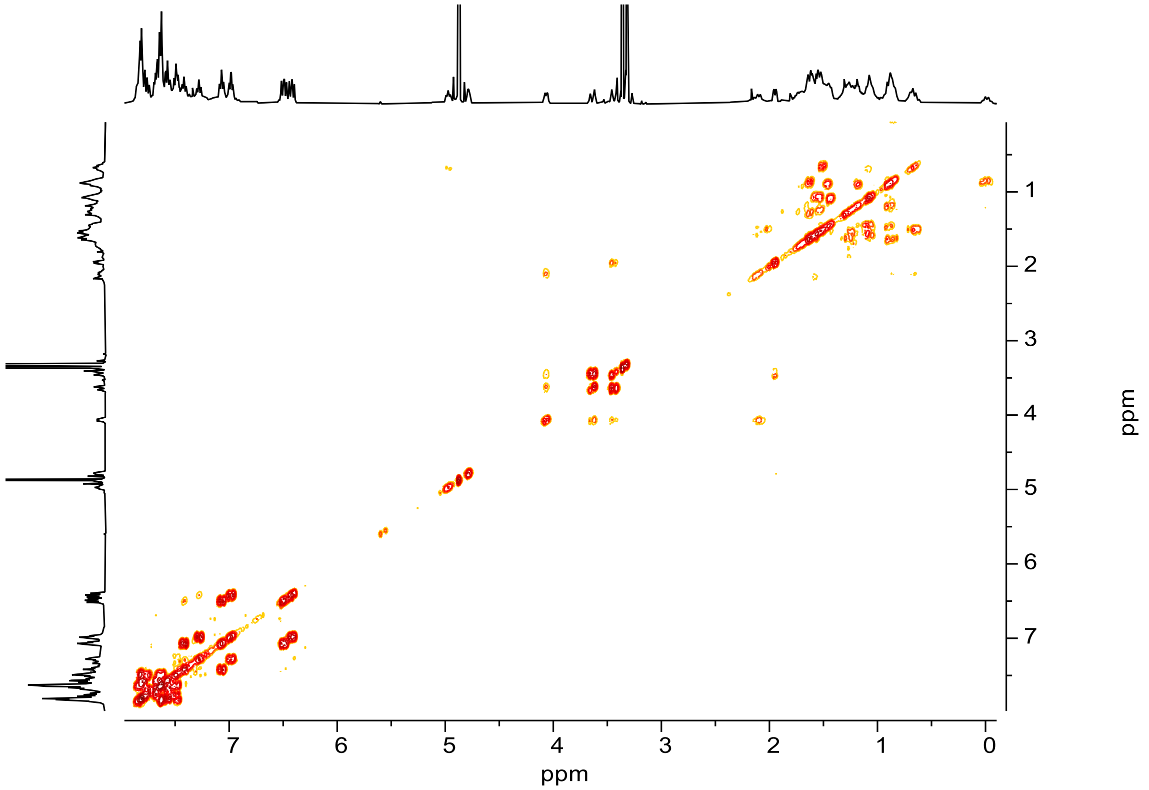

Figure S12. ^{1}H COSY NMR spectrum (24 °C) of **5** (10 mmol·L^{-1}) in MeOH-d_4.

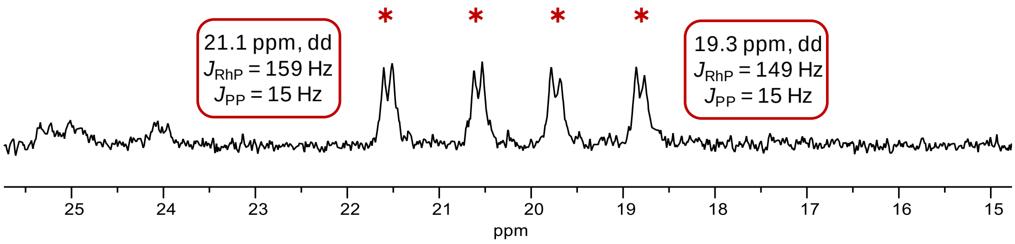

Figure S13. $^{31}P\{^{1}H\}$ NMR spectrum (162 MHz, 24 °C) of **5** (10 mmol·L^{-1}) in THF-d_8.

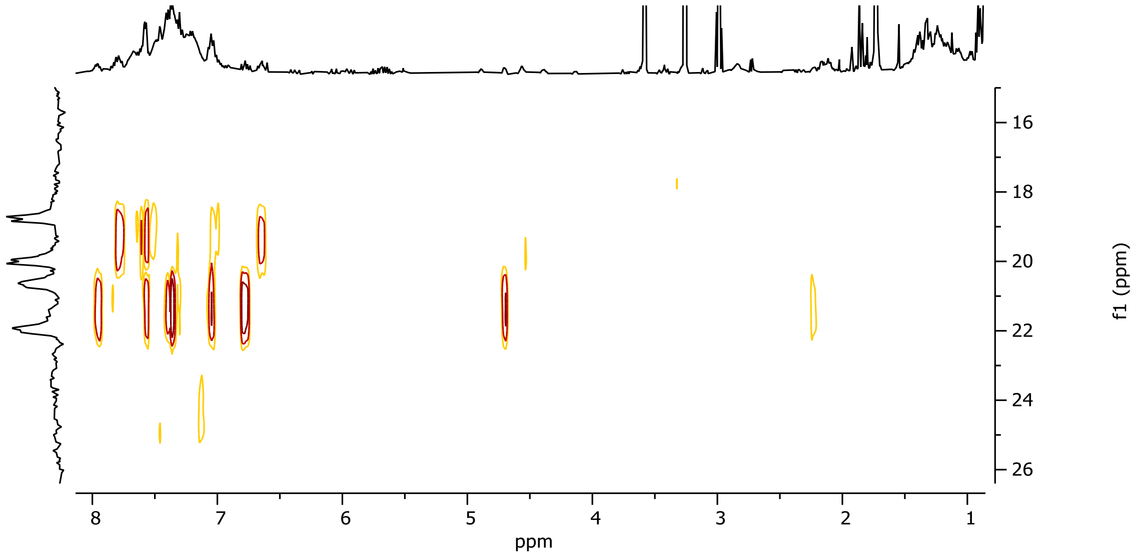

Figure S14. $^{1}H^{31}P\{^{1}H\}$ HMBC NMR spectrum (24 °C) of **5** (10 mmol·L^{-1}) in THF-d_8.

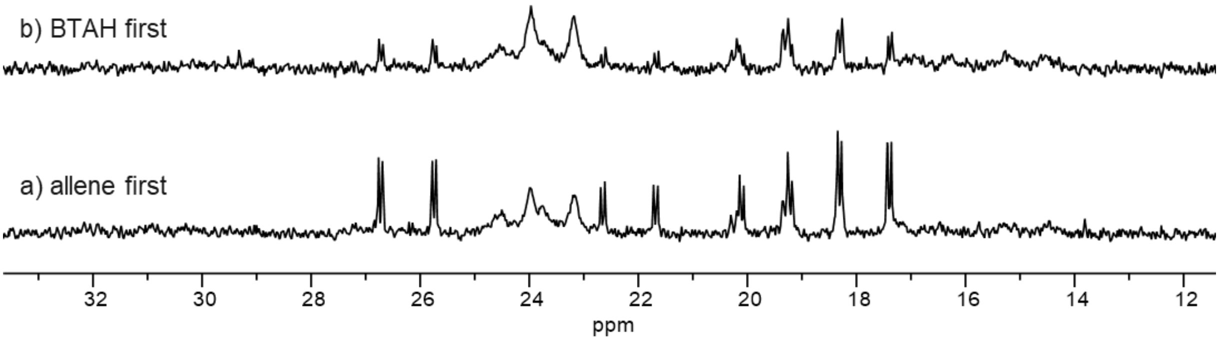

Figure S15. $^{31}P\{^{1}H\}$ NMR spectra (162 MHz, 50 °C) of 0.007 mmol [Rh(μ-Cl)(DPEPhos)]$_2$ (**1**) converted with 0.3 mmol BTAH and 0.4 mmol cyclohexyl allene in 1,2-DCE-d_4; **a)** pre-catalyst first converted with allene, **b)** pre-catalyst first converted with BTAH.

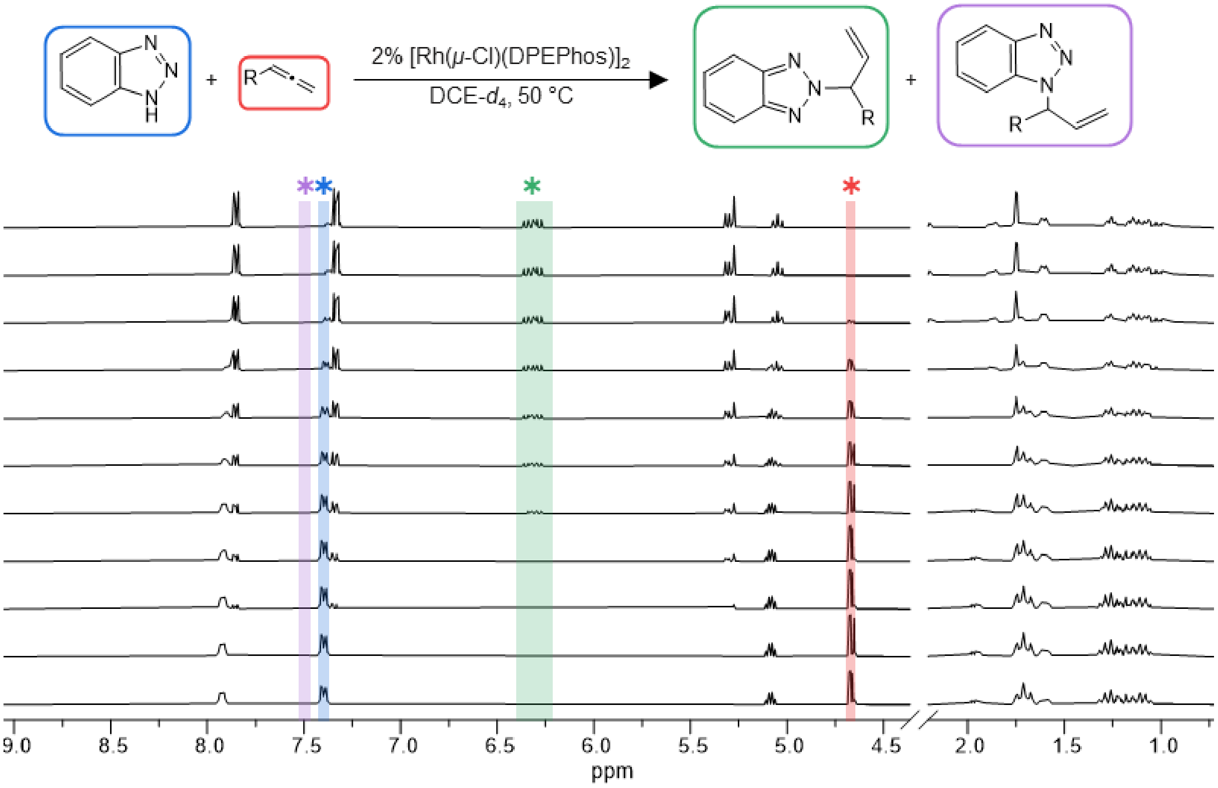

Figure S16. ^{1}H NMR spectroscopic (400 MHz) reaction monitoring at 50 °C of the conversion of 0.007 mmol [Rh(μ-Cl)(DPEPhos)]$_2$ (**1**) converted with 0.3 mmol BTAH and 0.4 mmol cyclohexyl allene in 1,2-DCE-*d*$_4$.

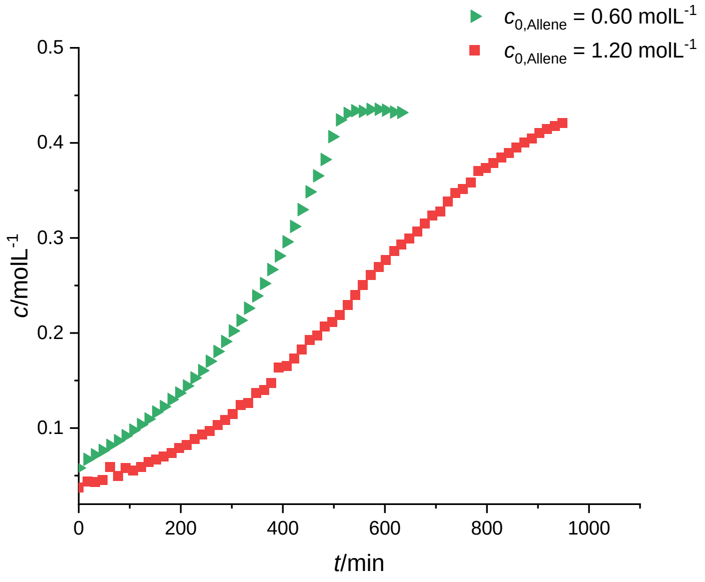

Figure S17. Concentration-time-plots of the conversion of 0.007 mmol [Rh(μ-Cl)(DPEPhos)]$_2$ (**1**), 0.3 mmol BTAH and 0.4 mmol (green squares) or 0.8 mmol (red triangles) cyclohexyl allene in 1,2-DCE-*d*$_4$ monitored by ^{1}H NMR spectroscopy (400 MHz, 50 °C).

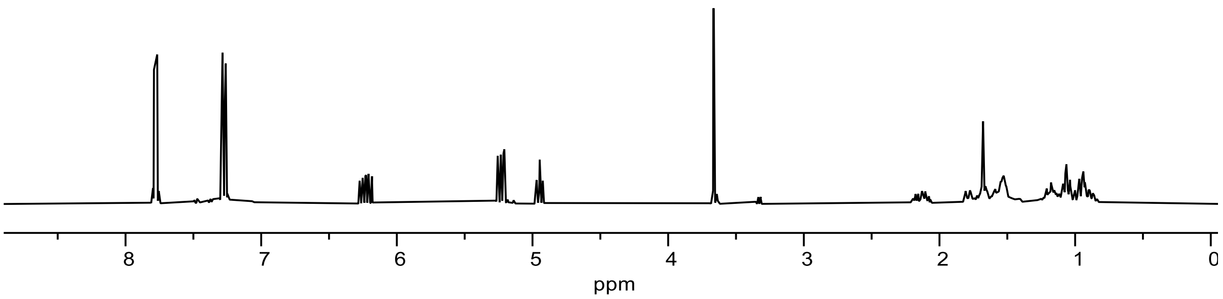

Figure S18. ^{31}H NMR spectrum (400 MHz, 24 °C) of 0.007 mmol [Rh(μ-Cl)(DPEPhos)]$_2$ (**1**) converted with 0.15 mmol BTAH and 0.2 mmol cyclohexyl allene in 1,2-DCE-d_4.

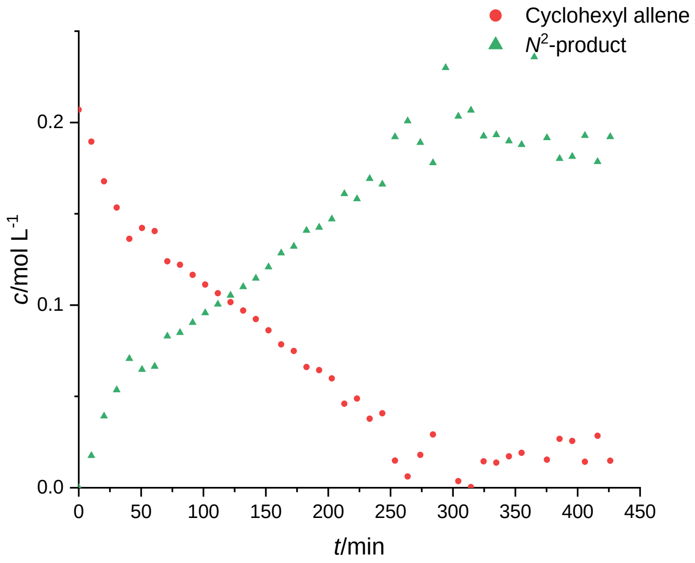

Figure S19. Corrected concentration-time-plots of the conversion of 0.007 mmol [Rh(μ-Cl)(DPEPhos)]$_2$ (**1**), 0.15 mmol BTAH and 0.2 mmol cyclohexyl allene in 1,2-DCE monitored by ^{1}H NMR spectroscopy (400 MHz, 24 °C).

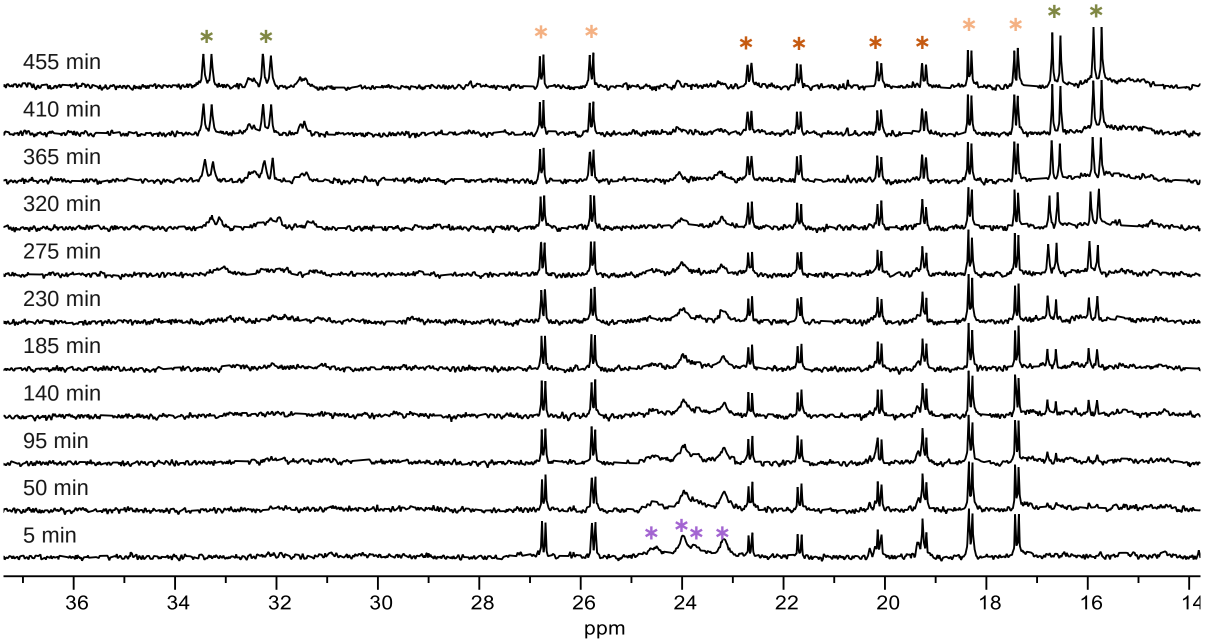

Figure S20. $^{31}P\{^1H\}$ NMR spectroscopic (162 MHz) reaction monitoring at 50 °C of 0.007 mmol [Rh(μ-Cl)(DPEPhos)]$_2$ (**1**) converted with 0.3 mmol BTAH and 0.4 mmol cyclohexyl allene in 1,2-DCE-*d*$_4$.

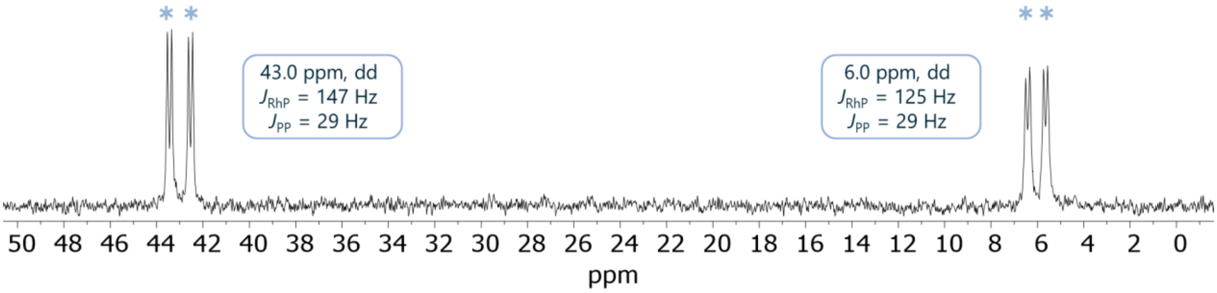

Figure S21. $^{31}P\{^1H\}$ NMR spectrum (162 MHz) of the conversion of 0.007 mmol [Rh(μ-Cl)(DPEPhos)]$_2$ (**1**) with 0.3 mmol *N*^{2}-product in 1,2-DCE-*d*$_4$.

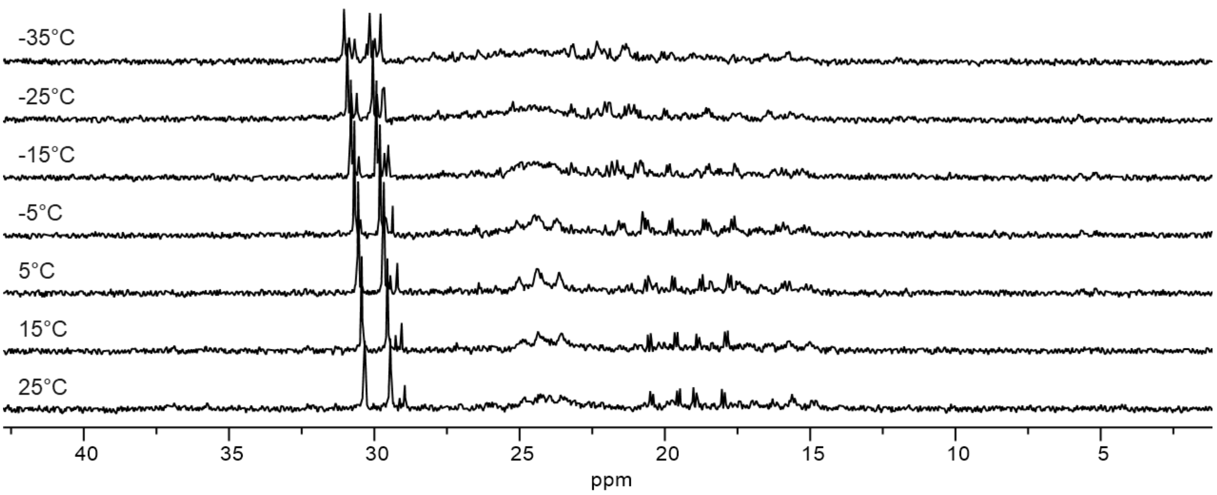

Figure S22. Variable temperature $^{31}P\{^{1}H\}$ NMR spectroscopy (162 MHz) of 0.007 mmol [Rh(μ-Cl)(DPEPhos)]$_2$ (**1**) converted with 0.13 mmol BTAH and 0.2 mmol cyclohexyl allene in 1,2-DCE-d_4.

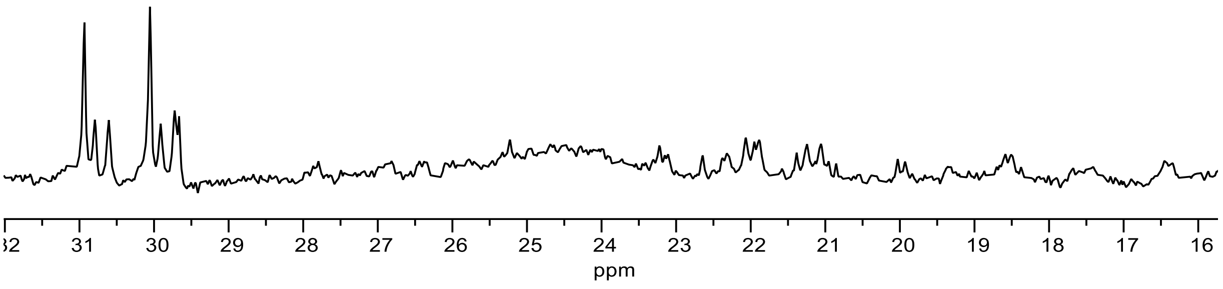

Figure S23. $^{31}P\{^{1}H\}$ NMR spectrum (162 MHz, -25 °C) of 0.007 mmol [Rh(μ-Cl)(DPEPhos)]$_2$ (**1**) converted with 0.13 mmol BTAH and 0.2 mmol cyclohexyl allene in 1,2-DCE-d_4.

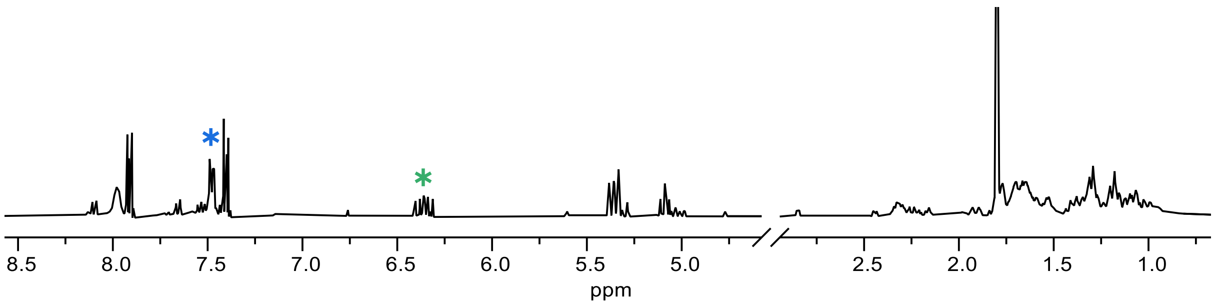

Figure S24. ^{1}H NMR spectrum (162 MHz, 25 °C) of 0.007 mmol [Rh(DPEPhos)]$_2$(BF$_4$)$_2$ (**1**) converted with 0.13 mmol BTAH and 0.2 mmol cyclohexyl allene in 1,2-DCE-d_4 (80 °C, 18 h).

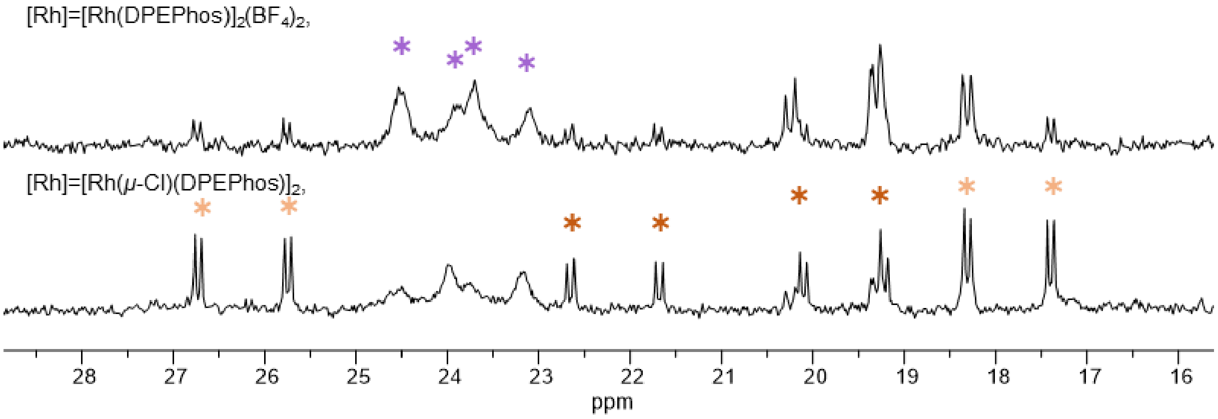

Figure S25. $^{31}P\{^{1}H\}$ NMR spectra (162 MHz, 50 °C) of the neutral complex [Rh(μ-Cl)(DPEPhos)]$_2$ (**1**) and the cationic complex [Rh(DPEPhos)]$_2$(BF$_4$)$_2$, each converted with catalytic amounts of BTAH and cyclohexyl allene in 1,2-DCE-d_4.

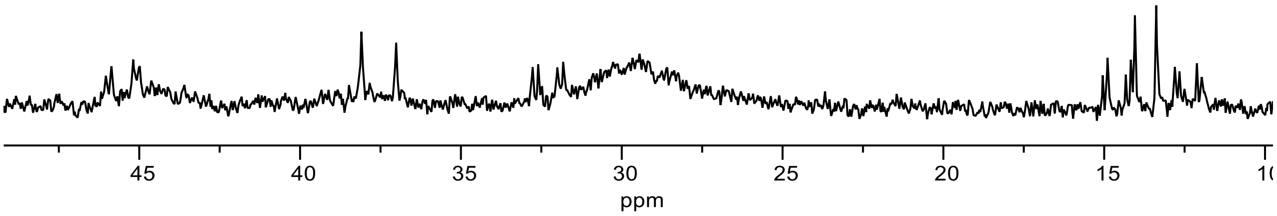

Figure S26. $^{31}P\{^{1}H\}$ NMR spectrum (162 MHz, 24 °C) of the conversion of [Rh(μ-Cl)(DPEPhos)]$_2$ (**1**) with 10 equivalents of PPTS in 1,2-DCE-d_4.

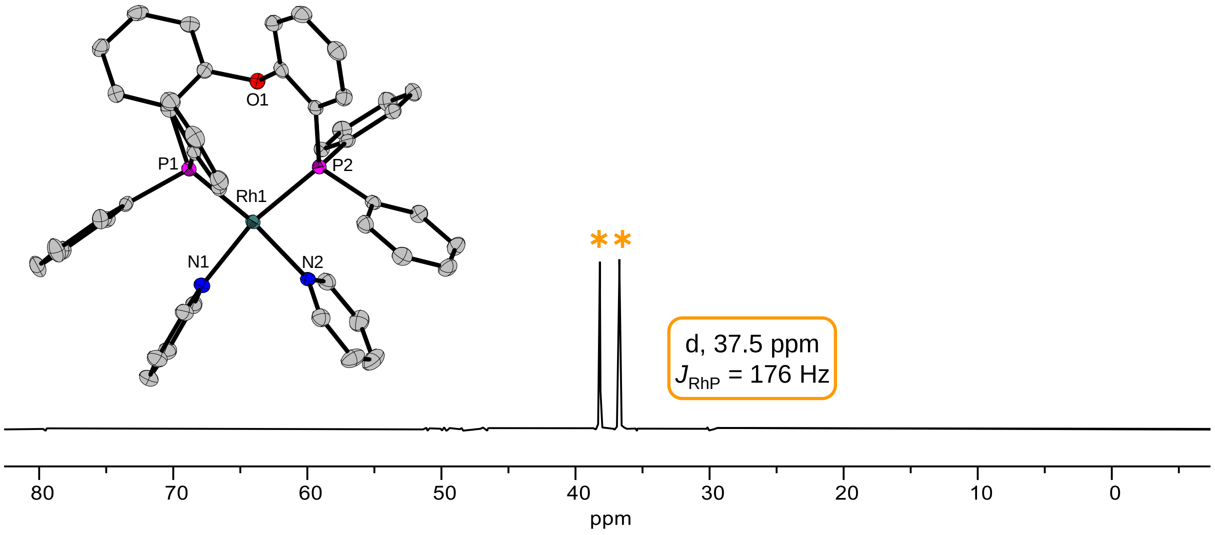

Figure S27. $^{31}P\{^{1}H\}$ NMR spectrum (122 MHz, 24 °C) of the conversion of [Rh(DPEPhos)(MeOH)]BF_4 with 50 equivalents of pyridine in MeOH-d_4.

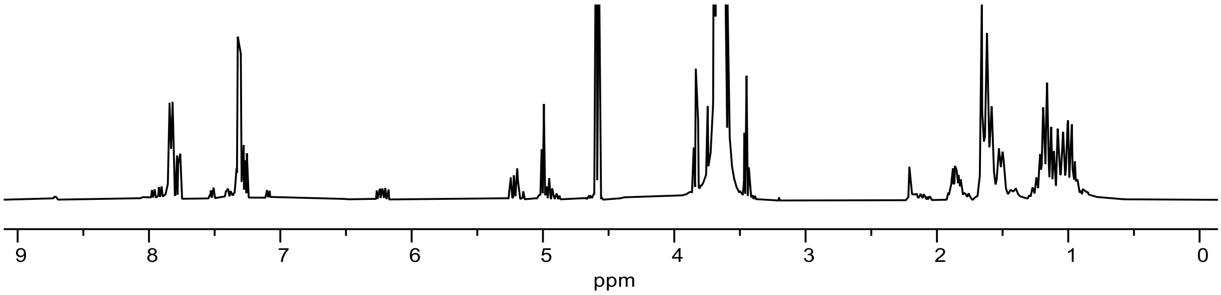

Figure S28. $^{31}P\{^{1}H\}$ NMR spectrum (162 MHz, 24 °C) of 0.007 mmol [Rh(μ-Cl)(DPEPhos)]$_2$ (**1**) and 0.01 mmol PPTS converted with 0.3 mmol BTAH and 0.4 mmol cyclohexyl allene in 1,2-DCE-d_4.

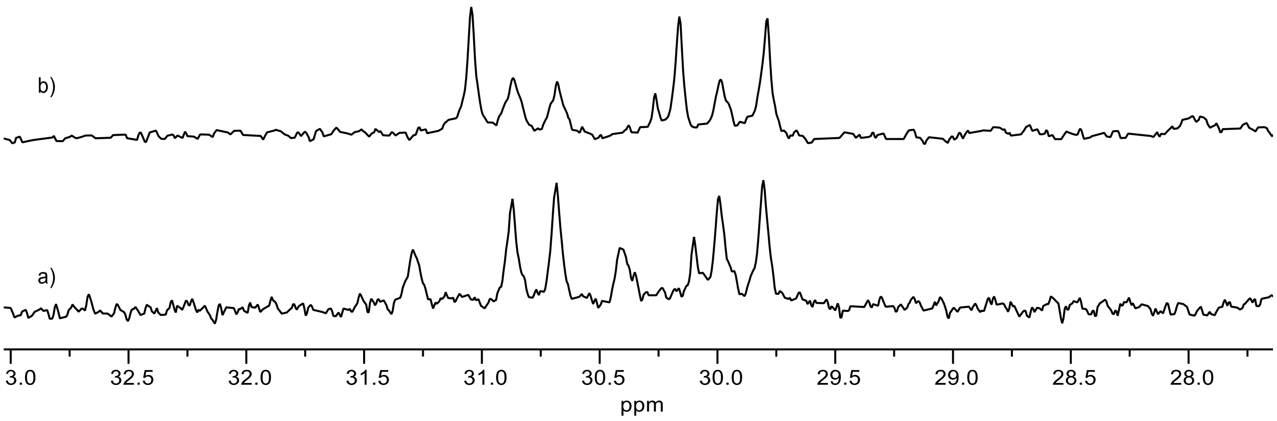

Figure S29. Comparison of the $^{31}P\{^{1}H\}$ NMR spectra of a) of 0.007 mmol [Rh(μ-Cl)(DPEPhos)]$_2$ (**1**) and 0.01 mmol PPTS converted with 0.3 mmol BTAH and 0.4 mmol cyclohexyl allene in 1,2-DCE-d_4 24 °C and b) of 0.007 mmol [Rh(μ-Cl)(DPEPhos)]$_2$ (**1**) converted with 0.13 mmol BTAH and 0.2 mmol cyclohexyl allene in 1,2-DCE-d_4 at -25 °C.

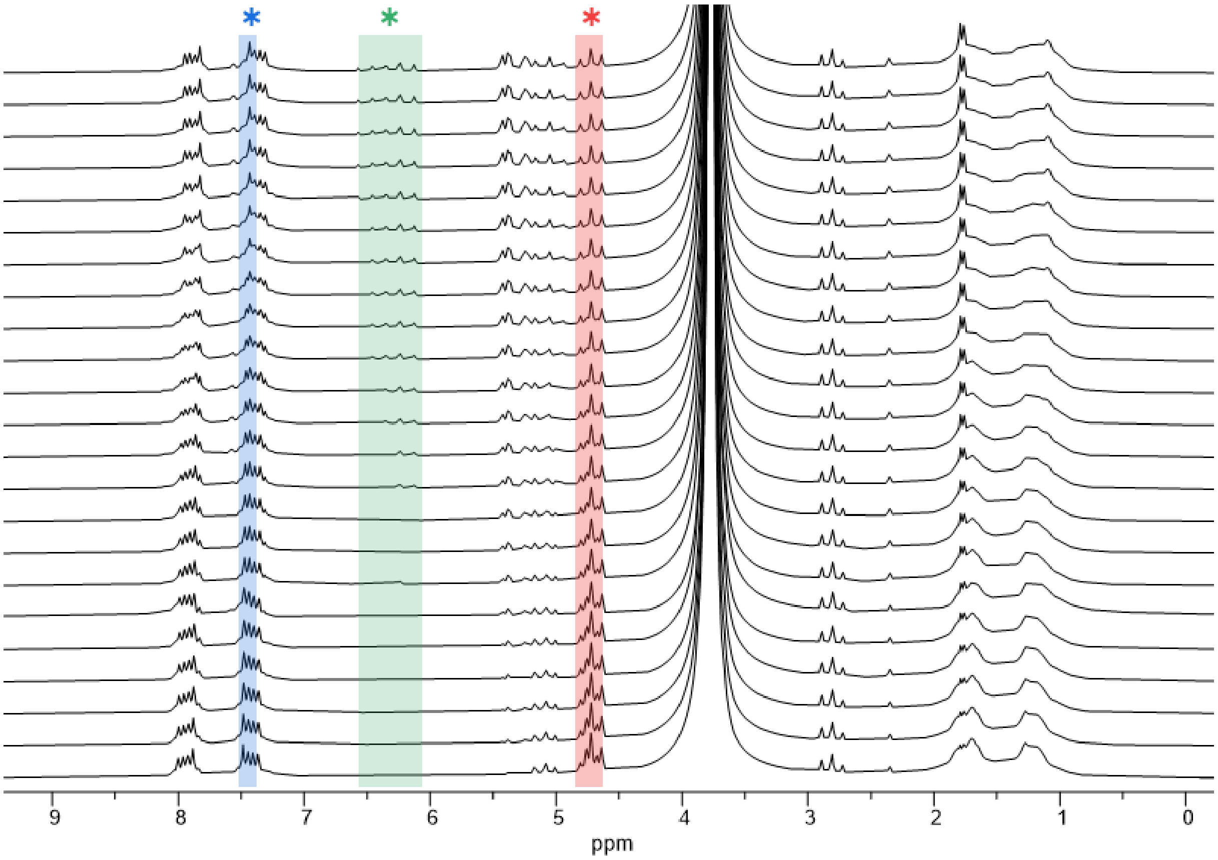

Figure S30. Selected ^{1}H NMR spectra (80 MHz, 25 °C) of the reaction of cyclohexyl allene (0.343 mmol·L^{-1}) * and BTAH (0.259 mmol·L^{-1}) * catalyzed by [Rh(μ-Cl)(DPEPhos)]$_2$ (**1**, 0.00615 mmol·L^{-1}) with PPTS (0.00917 mmol·L^{-1}) forming N^2-allyl benzotriazole * in 1,2-DCE.

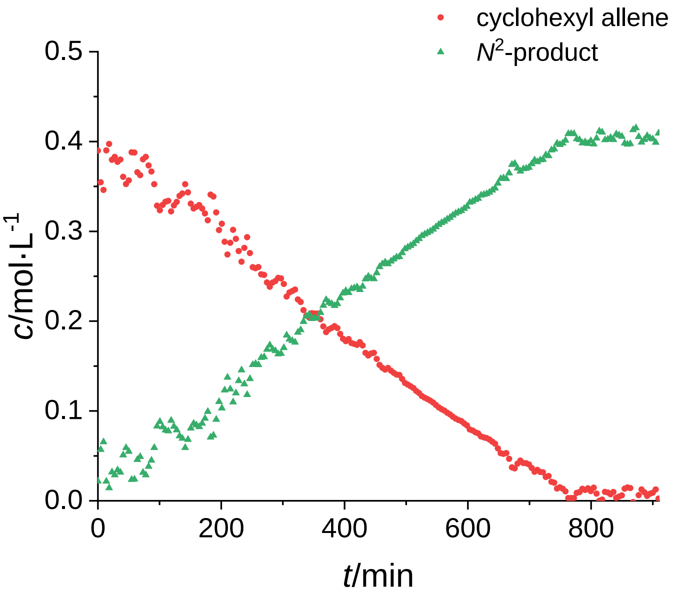

Figure S31. Conversion curve of the reaction of cyclohexyl allene (0.343 mmol·L^{-1}) * and BTAH (0.259 mmol·L^{-1}) * catalyzed by [Rh(μ-Cl)(DPEPhos)]$_2$ (**1**, 0.00615 mmol·L^{-1}) with PPTS (0.00917 mmol·L^{-1}) forming N^2-allyl benzotriazole * in 1,2-DCE. Concentrations were determined by ^{1}H NMR spectroscopy (80 MHz, 25 °C).

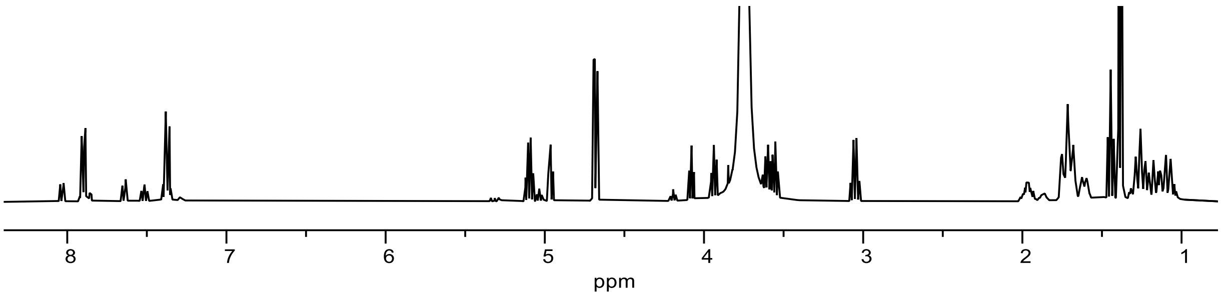

Figure S32. ^{31}P{^{1}H} NMR spectrum (162 MHz, 24 °C) of 0.007 mmol [Rh(μ-Cl)(DPEPhos)]$_2$ (**1**) and 0.14 mmol *Hünig's* base converted with 0.3 mmol BTAH and 0.4 mmol cyclohexyl allene in 1,2-DCE-d_4 after 18 h at 80 °C.

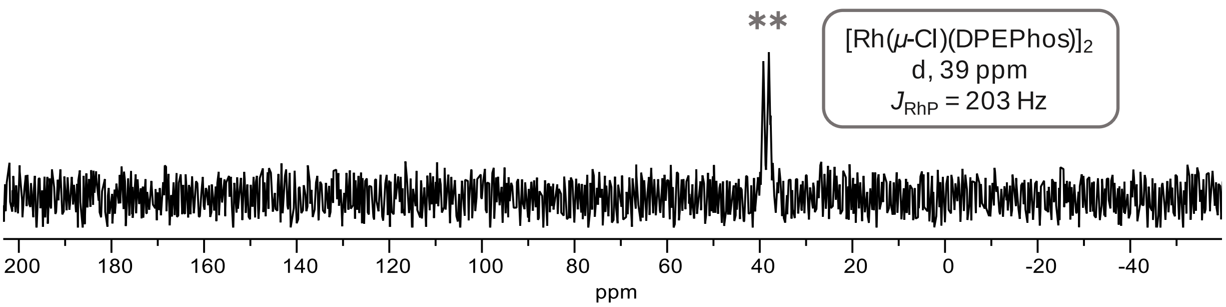

Figure S33. ^{31}P{^{1}H} NMR spectrum (162 MHz, 24 °C) of the conversion of [Rh(μ-Cl)(DPEPhos)]$_2$ (**1**) with 20 equivalents of *Hünig's* base in 1,2-DCE-d_4.

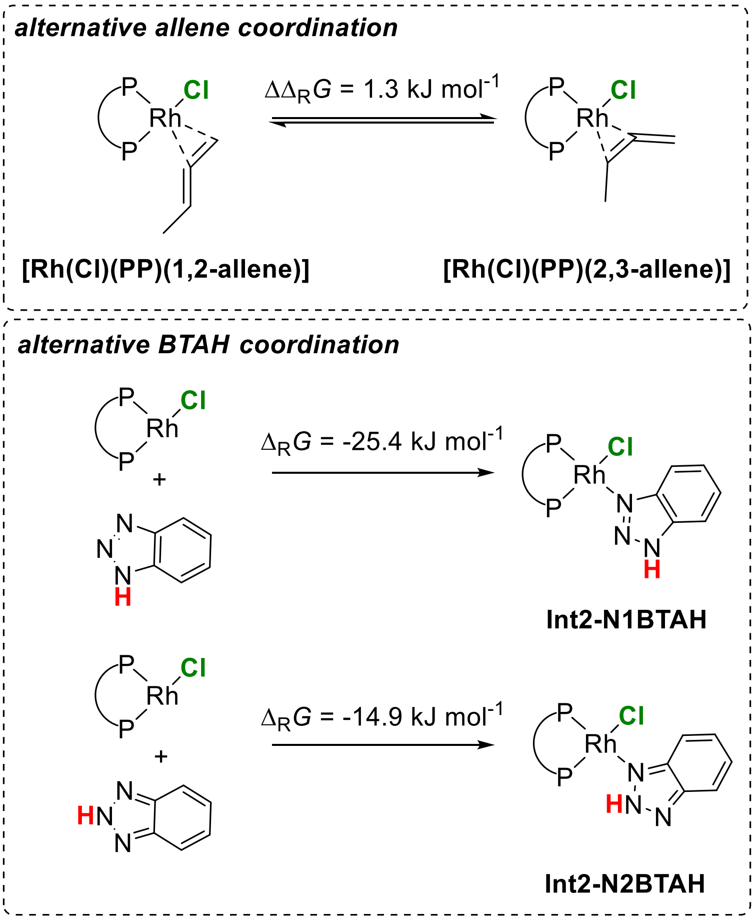

Figure S34. Alternative coordination modes of Me-allene and BTAH (DLNPO-CCSD(T)/def2-TZVP/SMD//B3LYP-D3/def2-SVPP).

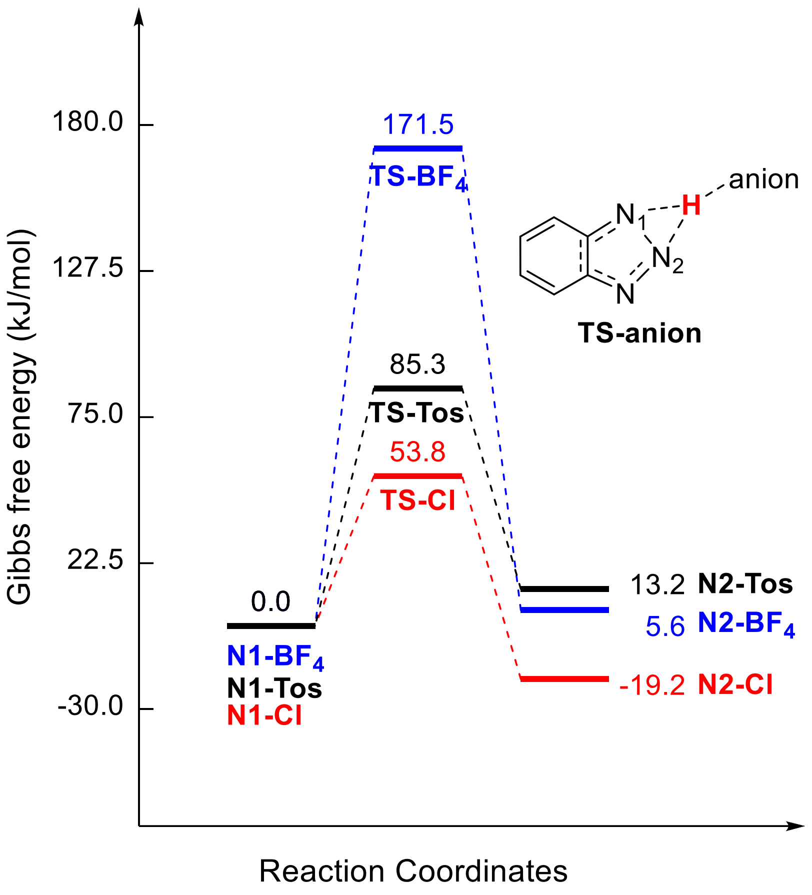

Figure S35. Comparison of the anion effect on the isomerization of free BTAH (DLNPO-CCSD(T)/def2-TZVP/SMD//B3LYP-D3/def2-SVPP).

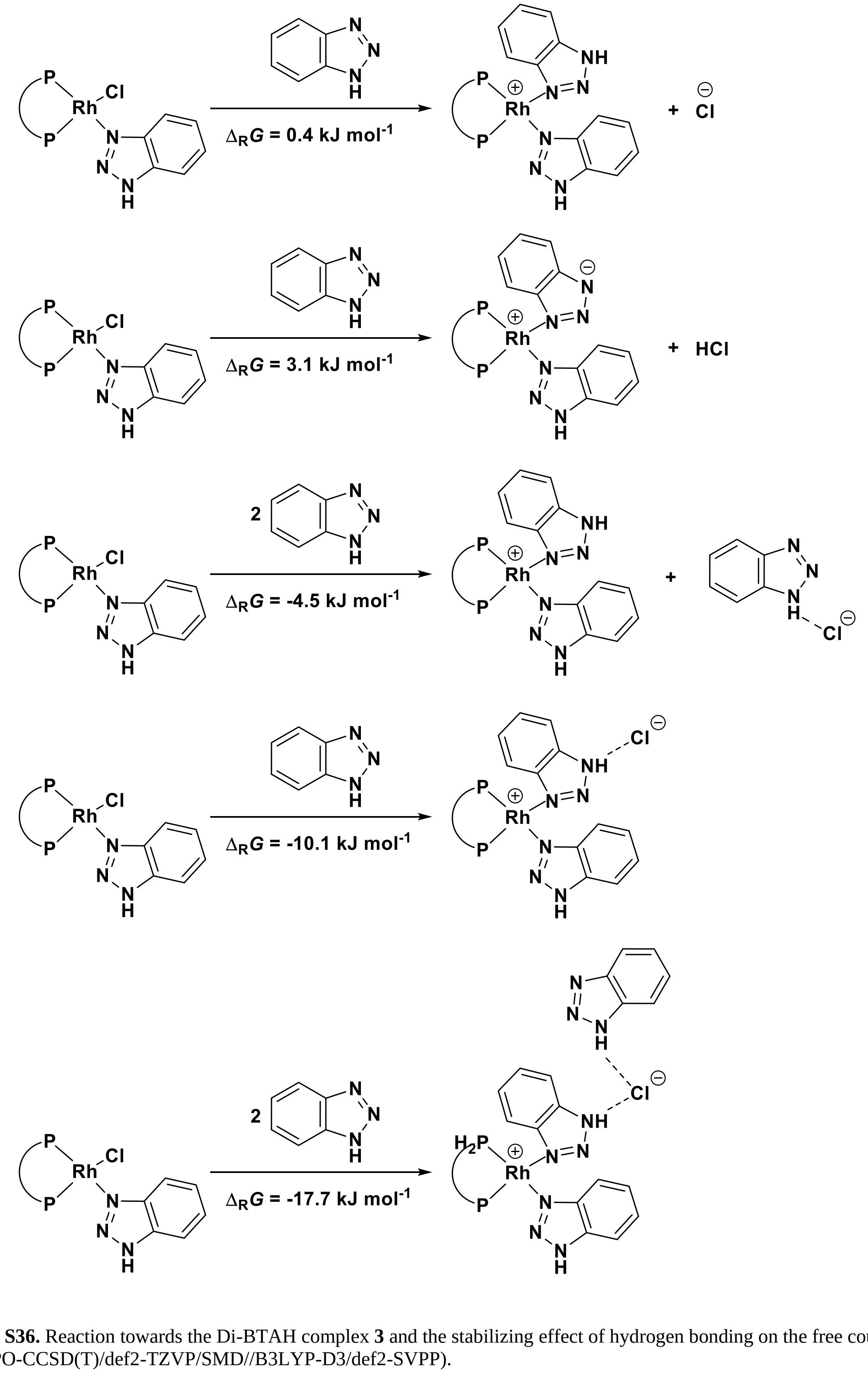

Figure S36. Reaction towards the Di-BTAH complex **3** and the stabilizing effect of hydrogen bonding on the free counterion (DLNPO-CCSD(T)/def2-TZVP/SMD//B3LYP-D3/def2-SVPP).

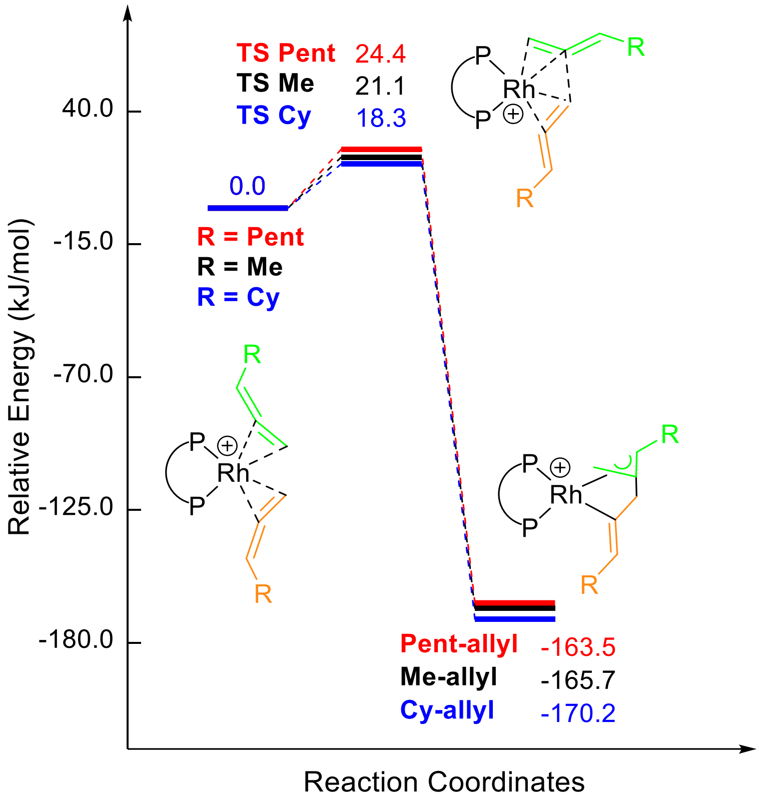

Figure S37. Reaction towards the Di-BTAH complex **3** and the stabilizing effect of hydrogen bonding on the free counterion (DLNPO-CCSD(T)/def2-TZVP/SMD//B3LYP-D3/def2-SVPP).

4 Crystallographic data

X-ray quality crystals were selected in Fomblin YR-1800 perfluoroether (Alfa Aesar) at low temperature. Diffraction data were collected at 150(2) K, except **[Rh(DPEPhos)(py)$_2$]BF$_4$** (110(2) K), on a Bruker Kappa APEX II Duo diffractometer using Mo-K$_\alpha$ radiation **2**, **6** and **[Rh(DPEPhos)(py)$_2$]BF$_4$** or Cu-K$_\alpha$ radiation **3** and **5**. The structures were solved by iterative (SHELXT)[5] or direct methods (SHELXS-97)[6] and refined by full matrix least square techniques against F^2 (SHELXL-2014)[7]. Semi-empirical absorption corrections were applied (SADABS/Bruker).[8] The non-hydrogen atoms were refined anisotropically. The hydrogen atoms, except for the hydrogens at the nitrogen atoms of **2** (determination and refinement from electron density), were placed in the theoretical positions and were refined by using the riding model.

Contributions of solvent molecules were removed in **2** and **6** from the diffraction data with PLATON/SQUEEZE.[9] DIAMOND (Crystal Impact GbR) was used for structure representations.

Crystallographic data (excluding structure factors) for the structures reported in this paper have been deposited at the Cambridge Crystallographic Data Centre. Copies of the data can be obtained free of charge on application to CCDC, 12 Union Road, Cambridge, CB21EZ, UK (fax: int. code + (1223) 336-033; e-mail: deposit@ccdc.cam.ac.uk

Table S1. Crystallographic details.

Compound	[Rh(Cl)(DPEPhos)(BTAH)] **(2)**	[Rh(DPEPhos)(BTAH)$_2$]BF$_4$ (**3**)
Chem. Formula	$C_{42}H_{32}ClN_3OP_2Rh$	$C_{48}H_{36}BF_4N_6OP_2Rh \cdot C_4H_8O$
Formula weight [g/mol]	795.00	1036.59
Color	orange	yellow
Crystal system	monoclinic	orthorhombic
Space group	*C*2/c	*P*na2$_1$
a [Å]	35.6716(11)	31.0251(12)
b [Å]	12.3668(4)	14.8965(6)
c [Å]	26.2984(8)	10.0834(4)
α [°]	90	90
β [°]	121.0810(10)	90
γ [°]	90	90
V [Å^3]	9935.9(5)	4660.2(3)
Z	8	4
$\rho_{calcd.}$ [g/cm^3]	1.063	1.477
μ [mm^{-1}]	0.490	4.158
Measured reflections	118972	32782
Independent reflections	12823	5315
Reflections with $I > 2\sigma(I)$	10038	5032
R_{int}	0.0463	0.0290
F(000)	3240	2120
No. of Parameters	451	544
$R_1(R[F^2>2\sigma(F^2)])$	0.0406	0.0280
w$R_2(F^2)$	0.1187	0.0746
GooF	1.039	1.054
Largest difference peak and hole (e/Å^{-3})	1.162/-0.649	0.385/-0.256
CCDC #	2258856	2258853

Table S1, continued. Crystallographic details.

Compound	$[Rh(DPEPhos)]_2(BF_4)_2$ **(6)**	$[Rh(DPEPhos)(py)_2]BF_4$
Chem. Formula	$C_{72}H_{56}B_2F_8P_4Rh_2$	$C_{46}H_{38}BF_4N_2OP_2Rh \cdot C_4H_8O$
Formula weight [g/mol]	1456.48	958.55
Color	red	yellow
Crystal system	triclinic	triclinic
Space group	$P\bar{1}$	$P\bar{1}$
a [Å]	12.6475(19)	10.3973(11)
b [Å]	13.501(2)	12.7013(14)
c [Å]	14.708(2)	16.9490(19)
α [°]	63.868(2)	90.576(3)
β [°]	65.488(2)	93.753(3)
γ [°]	72.515(2)	103.325(3)
V [Å^3]	2030.1(5)	2172.6(4)
Z	1	2
$\rho_{calcd.}$ [g/cm^3]	1.191	1.465
μ [mm^{-1}]	0.541	0.528
Measured reflections	104760	81711
Independent reflections	11413	8525
Reflections with $I > 2\sigma(I)$	10381	7249
R_{int}	0.0299	0.0588
$F(000)$	736	984
No. of Parameters	406	559
$R_1(R[F^2>2\sigma(F^2)])$	0.0257	0.0296
$wR_2(F^2)$	0.0689	0.0686
GooF	1.023	1.024
Largest difference peak and hole (e/Å^{-3})	0.716/-0.518	0.635/-0.335
CCDC #	2258855	2258854

Table S1, continued. Crystallographic details.

Compound	π-allyl-σ-vinyl-complex (**5**)
Chem. Formula	$C_{54}H_{56}BF_4P_2Rh$
Formula weight [g/mol]	972.64
Color	colourless
Crystal system	monoclinic
Space group	*C*2/c
a [Å]	25.6504(13)
b [Å]	12.6603(5)
c [Å]	30.1917(13)
α [°]	90
β [°]	102.201(5)
γ [°]	90
V [Å^3]	9583.0(8)
Z	8
$\rho_{calcd.}$ [g/cm^3]	1.348
μ [mm^{-1}]	3.948
Measured reflections	78602
Independent reflections	7626
Reflections with $I > 2\sigma(I)$	5967
R_{int}	0.0290
F(000)	4032
No. of Parameters	555
$R_1(R[F^2>2\sigma(F^2)])$	0.0607
w$R_2(F^2)$	0.1741
GooF	1.030
Largest difference peak and hole (e/Å^{-3})	1.567/-1.394
CCDC #	2258798

5 DFT calculations

5.1 General

We have chosen a multi-level quantum mechanical approach to answer the questions that have arisen during the experimental investigations as efficiently and accurately as possible.[10] The pre-optimizations and reaction path analysis were performed using xTB 6.5.1[11] (GFN2-xTB),[12] the obtained structures were re-optimized and checked at DFT level with Gaussian16[13] (B3LYP[14-19]-D3[20, 21]/def2-SVPP)[22], and single point calculations were performed to account for solvent correction with the same basis set (SMD,[23] 1,2-DCE) and to obtain more accurate electronic energies using ORCA[24-26] (DLPNO-CCSD(T)[27-31]/def2-TZVP, final notation DLPNO-CCSD(T)/def2-TZVP/SMD//B3LYP-D3/def2-SVPP). Reaction profiles were compiled using EnePro v1.6.[32]

5.2 Bond analysis

For a DFT analysis of the unusual rhodacycle **5**, its structure was first optimized using different methods, basis sets and with empirical dispersion and best agreement between computed and experimental geometries (Table S1, Table S2) was found for the BP86 method with a def2-SVPP basis set and GD3 empirical dispersion.

To better understand the binding situation of the π-allyl/σ-vinyl complex, an electron localization function (ELF)[33] analysis (Figure S38) and a combination of quantum theory of atoms in molecules (QT-AIM)[34] analysis has been performed, shown on the contour plots of the *Laplace* operator of electron density alongside the *Wiberg* bond binding indices (WBI)[35] (Figure S39), as well as natural bond orbital (NBO)[36-39] analysis to obtain the natural localized molecular orbitals (NLMO). In both, the experimental and the optimized structure, the central allyl carbon (C48) shows a shorter distance to Rh than the other allyl carbons (Table S2 and S3), raising the question whether all three allyl carbons bind to Rh. The QT-AIM analysis shows only one bond between Rh and the central allyl carbon C48. However, the electron density pointing from C47 and C58 toward Rh indicates polarized bonding between all allyl carbons and the metal center, in line with the WBI's (0.6). The delocalized double bond in the allyl unit is clearly indicated by the WBI of 1.29 and 1.33, respectively. In the same figure, the Rh-C50 σ-vinyl bond can be identified based on a bond critical point and a WBI of 0.99. A double bond (WBI = 1.84) is found between C50 and C51. Moreover, the Rh-C50 σ-bond and the binding of the allyl unit to the metal can be rationalized by NLMO analysis (Table S4). κ^3-P,O,P coordination of the DPEPhos ligand is characterized by a bond critical point between Rh and O1 (Figure S15), with low covalent character (WBI = 0.32), in line with a lone pair localized on O1 showing small contributions of a Rh d orbital (<2%, Table S4), indicating a strongly polarized weak Rh-O interaction.

Table S2. Optimized structures of **5** with different methods and a def2-SVPP basis set and the deviation from the geometry of the molecular structure.

parameter		molecular structure (Figure 6)	Optimized structures/deviation			
			M06/def2-SVPP	B3LYP/def2-SVPP	B98/def2-SVPP	BP86/def2-SVPP
bond length/Å	Rh1-P1	2.29	2.6%	3.5%	3.2%	2.2%
	Rh1-P2	2.34	3.5%	4.3%	3.7%	2.6%
	Rh1-O1	2.35	3.1%	2.9%	2.8%	2.4%
	Rh1-C47	2.27	0.7%	2.6%	1.7%	2.3%
	Rh1-C58	2.24	0.6%	1.6%	0.8%	1.4%
	Rh1-C48	2.14	0.0%	1.3%	0.8%	0.7%
	Rh1-C50	2.03	0.6%	0.2%	0.3%	0.4%
bond angle/°	P1-Rh1-P2	104.6	0.5%	0.8%	0.6%	0.9%
	C50-Rh1-O1	175.4	1.0%	0.2%	0.5%	0.3%
	C48-Rh1-C50	67.4	0.4%	0.4%	0.1%	0.1%
	C47-C48-C58	119.2	0.2%	0.8%	0.5%	0.5%
	**total deviation**		**1.21%**	**1.68%**	**1.36%**	**1.27%**

Table S3. Optimized structures of **5** with different methods, basis sets and empirical dispersion and the deviation from the geometry of the molecular structure.

**parameter**		**molecular structure (Figure 6)**	**Optimized structures/deviation**					
			**M06/def2-SVPP**	**M06+GD3/def2-SVPP**	**BP86/def2-SVPP**	**BP86+GD3/def2-SVPP**	**BP86/def2-TZVP**	**BP86+GD3/def2-TZVP**
**bond length/Å**	Rh1-P1	2.29	2.6%	2.4%	2.2%	0.7%	1.7%	0.0%
	Rh1-P2	2.34	3.5%	3.2%	2.6%	0.2%	1.6%	0.6%
	Rh1-O1	2.35	3.1%	3.0%	2.4%	1.6%	2.3%	1.5%
	Rh1-C47	2.27	0.7%	0.9%	2.3%	0.9%	3.2%	1.5%
	Rh1-C58	2.24	0.6%	0.6%	1.4%	1.2%	1.2%	1.2%
	Rh1-C48	2.14	0.0%	0.0%	0.7%	0.4%	0.7%	0.4%
	Rh1-C50	2.03	0.6%	0.9%	0.4%	0.5%	0.2%	0.7%
**bond angle/°**	P1-Rh1-P2	104.6	0.5%	0.9%	0.9%	1.6%	0.9%	1.3%
	C50-Rh1-O1	175.4	1.0%	1.3%	0.3%	0.5%	0.8%	0.8%
	C48-Rh1-C50	67.4	0.4%	0.4%	0.1%	0.6%	0.1%	0.6%
	C47-C48-C58	119.2	0.2%	0.1%	0.5%	0.6%	0.9%	0.8%
**total deviation**			**1.21%**	**1.25%**	**1.27%**	**0.80%**	**1.24%**	**0.86%**

Table S4. Optimized structures of **5** with B3LYP/def2-SVPP and GFN2-xTB and the deviation from the geometry of the molecular structure.

**parameter**		**molecular structure (Figure 6)**	**Optimized structures/deviation**	
			**B3LYP/def2-SVPP**	**GFN2-xTB**
**bond length/Å**	Rh1-P1	2.29	3.5%	2.2%
	Rh1-P2	2.34	4.3%	0.5%
	Rh1-O1	2.35	2.9%	6.9%
	Rh1-C47	2.27	2.6%	44.5%
	Rh1-C58	2.24	1.6%	5.9%
	Rh1-C48	2.14	1.3%	15.8%
	Rh1-C50	2.03	0.2%	0.5%
**bond angle/°**	P1-Rh1-P2	104.6	0.8%	0.2%
	C50-Rh1-O1	175.4	0.2%	0.6%
	C48-Rh1-C50	67.4	0.4%	7.7%
	C47-C48-C58	119.2	0.8%	6.8%
	**total deviation**		**1.68%**	**8.33%**

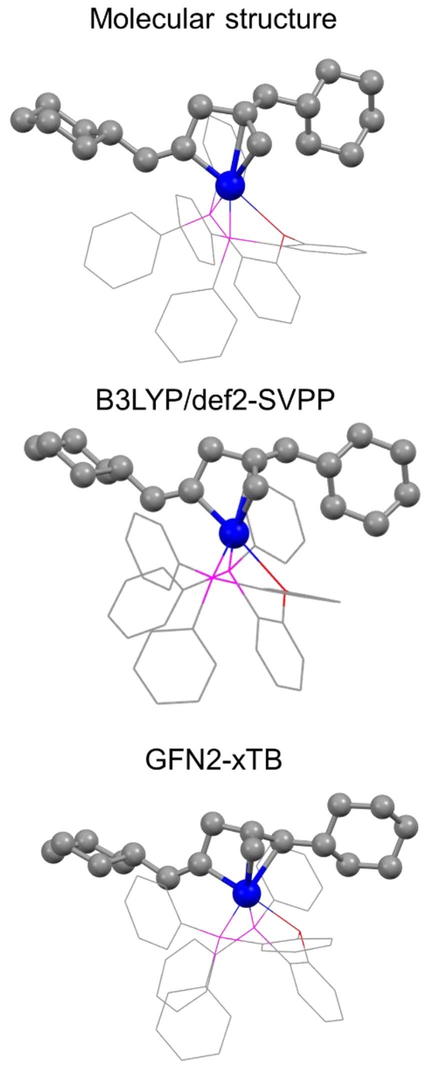

Numbering scheme for complex 5

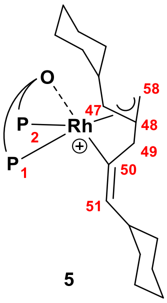

5

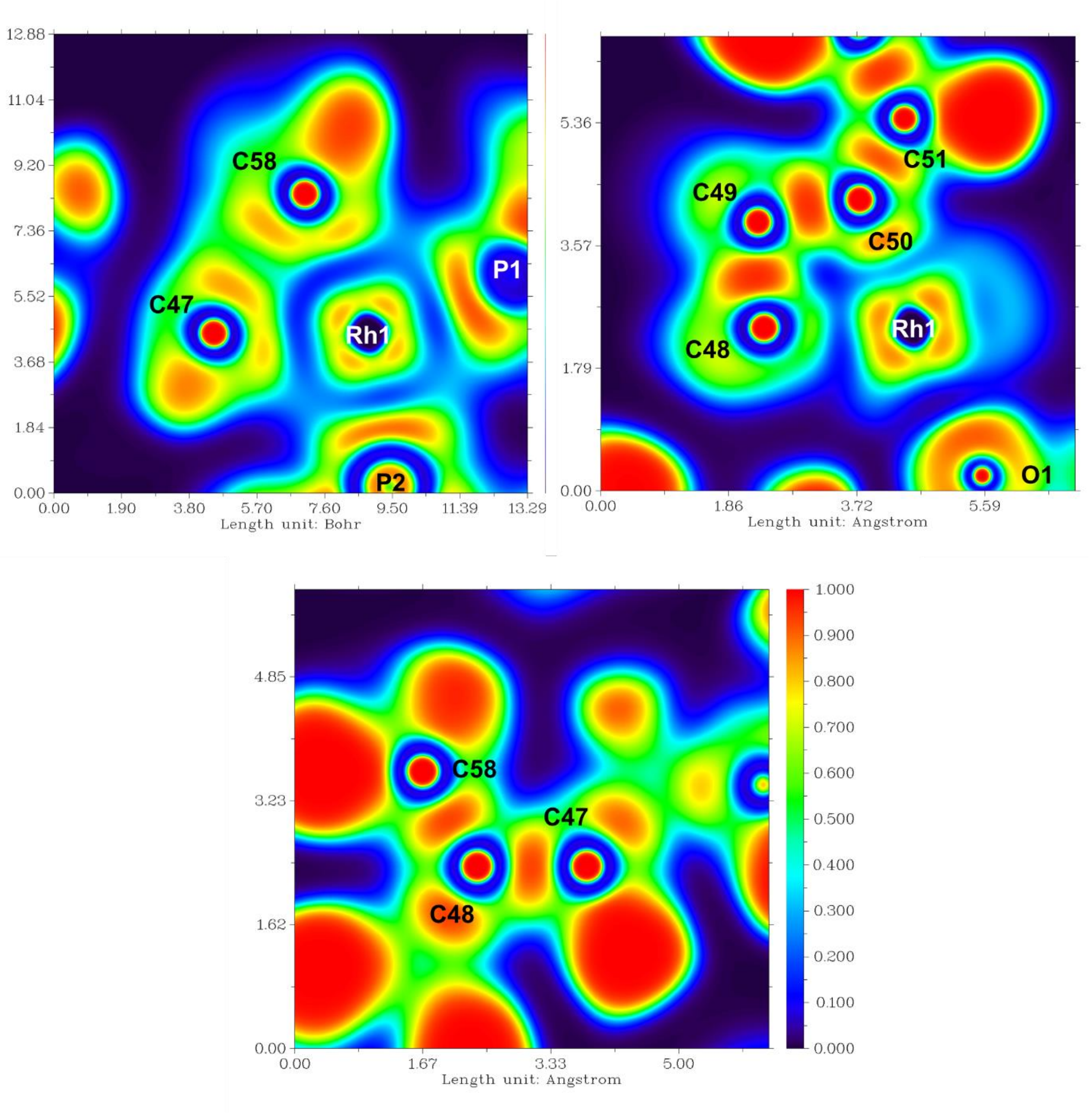

Figure S38. Electron Localization Function (ELF) analysis of **5** in three different planes; a) C47-Rh1-C58, b) C48-Rh1-C50, c) C47-C48-C58. Geometry optimized by BP86+GD3/def2-SVPP.

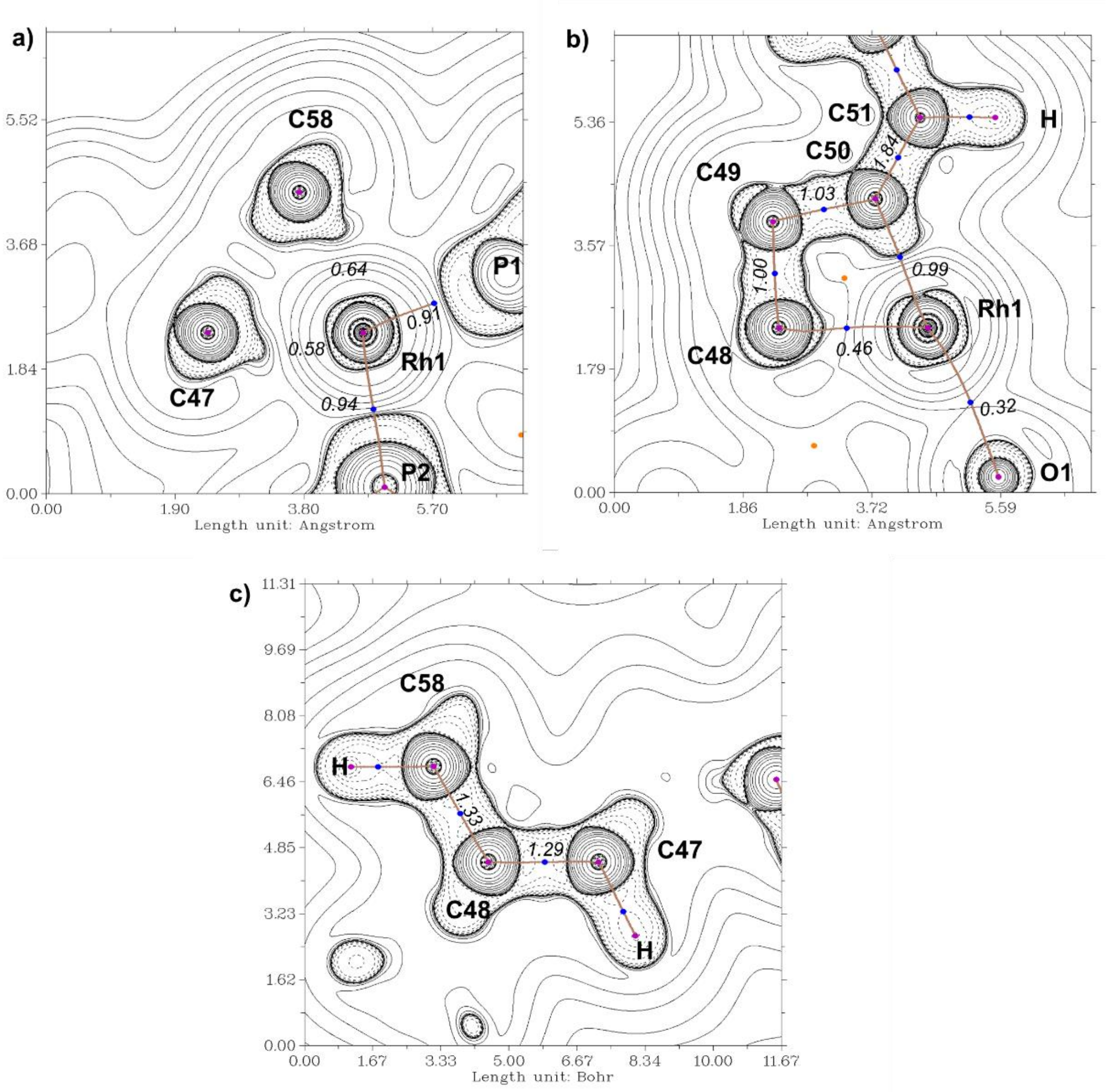

Figure S39. Contour plots of the *Laplace* operators of the electron density $\nabla^2 r$ of **5** in 3 different planes; a) C47-Rh1-C58, b) C48-Rh1-C50, c) C47-C48-C58. Dashed lines show negative values (local charge concentration), solid lines show positive values (local charge depletion). The *Laplace* plot is overlaid with the molecular diagram from the QT-AIM analysis and the *Wiberg* binding indices (italics). Brown lines indicate bond paths, blue dots correspond to critical bond points, and orange dots indicate critical ring points. Geometry optimized by BP86+GD3/def2-SVPP.

5.3 TD-UV-vis computation

The UV-vis spectrum of complex **5** in MeOH shows no absorption maxima in the visible region of the spectrum (Figure S41). Assignment of the transitions was done using time-dependent DFT (TD-DFT) UV-vis computations.

TD-DFT calculations were performed to predict UV-vis absorption spectra on Gaussian 16 using the B3LYP method, a def2-SVPP basis set and a PCM solvent model for methanol considering 40 excited states. The geometry of the unoptimized molecular structure was used for this calculation. The calculated data were plotted with a half-width of 2000 cm^{-1} and normalized to the maximum. The corresponding simulated UV-vis spectrum and the oscillator strength of the excited states is shown in Figure S40 and the first 20 excited states are reported in the following.

The calculated and experimental spectra are in very good agreement, with the calculated absorption maximum at 265 nm. According to the charge density difference analysis of the first exited states, all absorption bands correspond to metal-to-ligand charge transitions together with prominent ligand-to-ligand charge transfer.

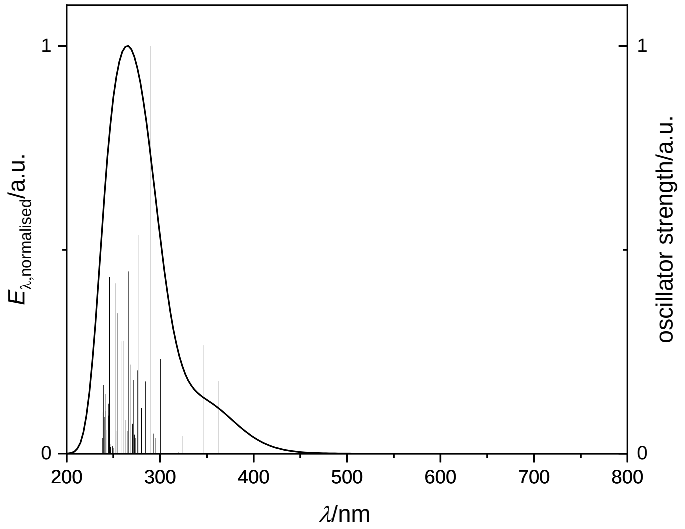

Figure S40. Normalized calculated UV-vis spectrum and the oscillator strength of **5**.

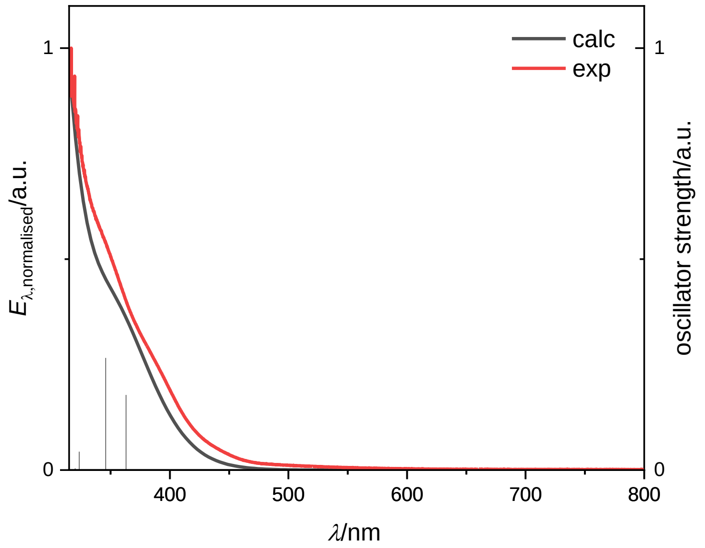

Figure S41. Normalized experimental (ca. 0.05 mmol in 16 mL MeOH) and theoretical (B3LYP/def2-SVPP/PCM=MeOH) UV-vis spectrum of **5**.

Excitation energies and oscillator strengths:

Excited State 1: Singlet-A 3.4155 eV 363.00 nm f=0.0225 <S**2>=0.000

217 ->218 -0.34450

217 ->219 0.39544

217 ->221 -0.37051

217 ->229 0.16957

This state for optimization and/or second-order correction.

Total Energy, E(TD-HF/TD-DFT) = -2957.09542075

Copying the excited state density for this state as the 1-particle RhoCI density.

Excited State 2: Singlet-A 3.5842 eV 345.92 nm f=0.0336 <S**2>=0.000

217 ->218 -0.35227

217 ->219 -0.29524

217 ->220 0.46777

217 ->221 -0.11368

Excited State 3: Singlet-A 3.8329 eV 323.48 nm f=0.0055 <S**2>=0.000

217 ->218 0.39788

217 ->219 0.32647

217 ->220 0.46865

Excited State 4: Singlet-A 3.8737 eV 320.06 nm f=0.0005 <S**2>=0.000

217 ->218 -0.28748

217 ->219 0.32034

217 ->220 0.14120

217 ->221 0.52647

Excited State 5: Singlet-A 4.1265 eV 300.46 nm f=0.0294 <S**2>=0.000

213 ->219 -0.10623

213 ->220 0.10749

215 ->219 0.34036

215 ->220 -0.32628

216 ->218 0.16258

216 ->219 0.17421

216 ->220 -0.27169

Excited State 6: Singlet-A 4.2059 eV 294.78 nm f=0.0048 <S**2>=0.000

217 ->222 0.69766

Excited State 7: Singlet-A 4.2361 eV 292.68 nm f=0.0062 <S**2>=0.000

213 ->219 0.10499

213 ->221 -0.12457

215 ->218 0.20932

215 ->219 -0.21038

215 ->221 0.19586

215 ->229 -0.10338

216 ->218 0.33725

216 ->219 -0.15746

216 ->220 -0.10711

216 ->221 0.24271

216 ->229 -0.10806

Excited State 8: Singlet-A 4.2852 eV 289.33 nm f=0.1263 <S**2>=0.000

207 ->219 0.12088

213 ->219 0.11148

215 ->218 0.14662

215 ->221 0.15485

216 ->218 -0.20462

216 ->219 0.36498

216 ->220 -0.14957

216 ->221 -0.19199

217 ->219 0.14670

217 ->221 -0.12159

Excited State 9: Singlet-A 4.3577 eV 284.51 nm f=0.0224 <S**2>=0.000

217 ->223 0.61130

217 ->224 -0.30735

Excited State 10: Singlet-A 4.4256 eV 280.15 nm f=0.0143 <S**2>=0.000

217 ->223 0.33263

217 ->224 0.58542

Excited State 11: Singlet-A 4.4848 eV 276.46 nm f=0.0679 <S**2>=0.000

213 ->220 0.10835

215 ->218 0.37017

215 ->220 -0.13296

216 ->218 -0.19790

216 ->219 -0.10210

216 ->220 0.24072

217 ->224 0.17777

217 ->225 0.13276

217 ->226 -0.22997

Excited State 12: Singlet-A 4.4930 eV 275.95 nm f=0.0258 <S**2>=0.000

207 ->218 -0.12709

207 ->219 0.10339

207 ->221 -0.17385

212 ->221 -0.13368

213 ->218 -0.13613

213 ->219 0.12967

213 ->221 -0.17625

215 ->218 -0.18057

215 ->219 0.22937

215 ->220 -0.12381

216 ->218 -0.22035

216 ->219 -0.16073

216 ->220 0.14678

Excited State 13: Singlet-A 4.5261 eV 273.93 nm f=0.0048 <S**2>=0.000

213 ->219 0.12029

213 ->220 -0.14023

215 ->219 0.15300

216 ->218 0.33672

216 ->220 0.24740

216 ->221 -0.11050

217 ->225 0.27952

217 ->226 -0.20505

217 ->227 -0.12125

217 ->229 -0.10430

Excited State 14: Singlet-A 4.5481 eV 272.61 nm f=0.0059 <S**2>=0.000

216 ->218 -0.12592

216 ->219 -0.10038

216 ->220 -0.23411

217 ->225 0.60536

217 ->226 0.12480

Excited State 15: Singlet-A 4.5696 eV 271.32 nm f=0.0229 <S**2>=0.000

215 ->218 0.16181

215 ->220 0.13048

216 ->218 0.12399

216 ->219 0.31424

216 ->220 0.33855

217 ->225 0.11510

217 ->226 0.38806

217 ->229 0.12948

Excited State 16: Singlet-A 4.5839 eV 270.48 nm f=0.0093 <S**2>=0.000

215 ->218 0.36107

215 ->219 0.36042

215 ->220 0.35690

215 ->221 -0.11926

216 ->219 -0.15913

216 ->220 -0.15346

Excited State 17: Singlet-A 4.6275 eV 267.93 nm f=0.0276 <S**2>=0.000

215 ->219 0.11140

215 ->220 0.22586

216 ->218 -0.16517

216 ->219 0.23063

216 ->221 0.50348

217 ->226 -0.24337

Excited State 18: Singlet-A 4.6531 eV 266.46 nm f=0.0564 <S**2>=0.000

213 ->218 0.10777

213 ->219 0.13453

213 ->220 -0.16245

215 ->218 0.10279

215 ->220 -0.22649

216 ->218 -0.15259

216 ->221 0.22558

217 ->226 0.34527

217 ->227 -0.17296

217 ->229 -0.13156

Excited State 19: Singlet-A 4.6819 eV 264.82 nm f=0.0071 <S**2>=0.000

213 ->220 -0.10792

215 ->218 0.11569

215 ->220 -0.18807

217 ->226 -0.14751

217 ->227 0.38216

217 ->228 0.23487

217 ->229 0.33446

Excited State 20: Singlet-A 4.7077 eV 263.36 nm f=0.0104 <S**2>=0.000

217 ->227 0.51680

217 ->228 -0.22594

217 ->229 -0.36567

Table S5. Orbital representation of selected excited states of the TD-UV-vis computation of **5**.

Excited state 1

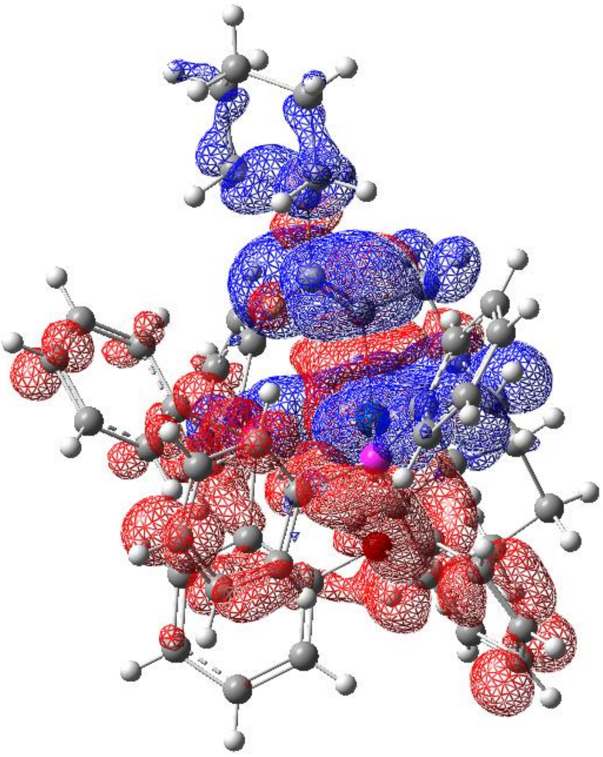

Excited state 2

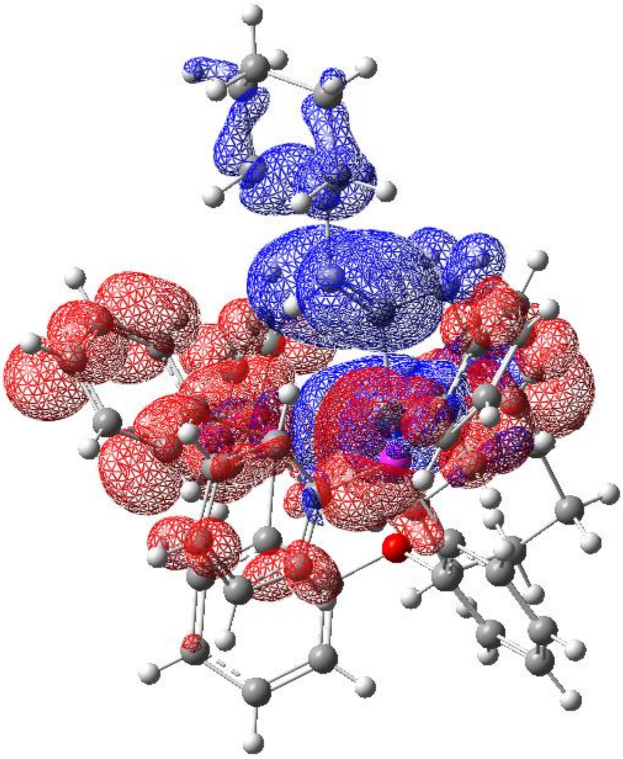

Excited state 3

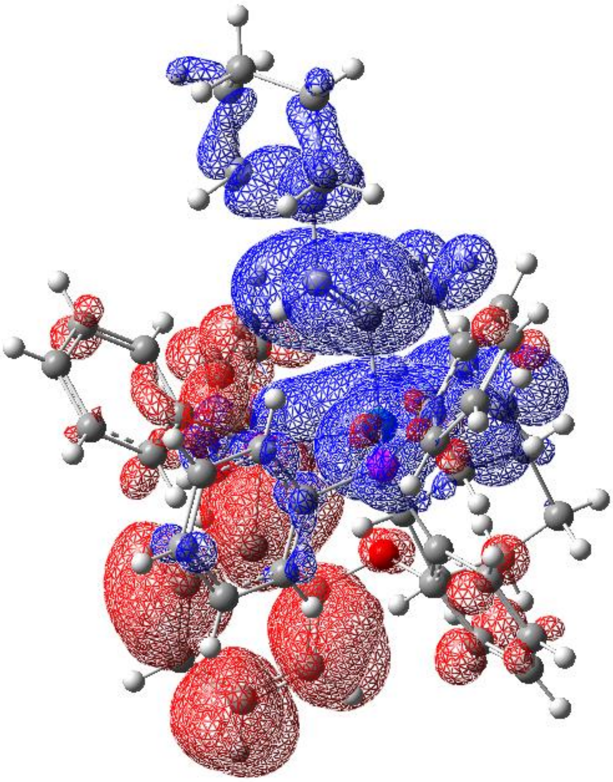

Excited state 8

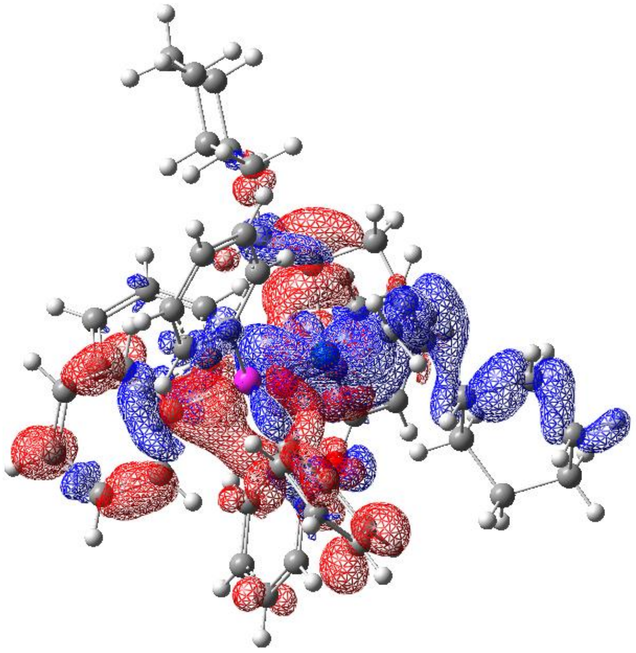

Excited state 18

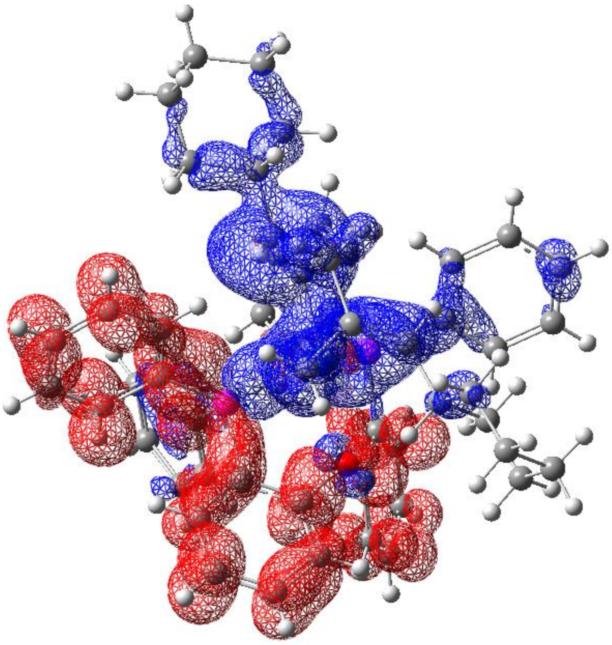

6 References

(1) Möller, S.; Drexler, H.-J.; Heller, D. Two precatalysts for application in propargylic CH activation. *Acta Crystallogr. C* **2019**, *75* (10), 1434-1438.
(2) Heller, D.; de Vries, A. H. M.; de Vries, J. G. Kinetics of Homogeneous Hydrogenations: Measurement and Interpretation. In *Handbook of Homogeneous Hydrogenation*, de Vries, J. G., Elsevier, C. J. Eds.; Vol. 1; Wiley-VCH, 2007; pp 257-293.
(3) Alberico, E.; Möller, S.; Horstmann, M.; Drexler, H.-J.; Heller, D. Activation, Deactivation and Reversibility Phenomena in Homogeneous Catalysis: A Showcase based on the Chemistry of Rhodium/Phosphine Catalysts. *Catalysts* **2019**, *9* (7), 582-627.
(4) Möller, S.; Kubis, C.; Drexler, H.-J.; Alberico, E.; Heller, D. Investigations into the mechanism of the in situ formation of neutral dinuclear rhodium complexes. *J. Organomet. Chem.* **2019**, *904*, 121002-121006.
(5) Sheldrick, G. M. *Acta Cryst. A* **2015**, *71*, 3-8.
(6) Sheldrick, G. M., *SHELXS 97, Program for the Solution of Crystal Structure*; University of Göttingen: Göttingen, 1990.
(7) Sheldrick, G. M. *Acta Cryst. C* **2015**, *71*, 3-8.
(8) Sheldrick, G. M., *SADABS Version 2*; University of Göttingen: Göttingen, 2004.
(9) Spek, T. *Acta Cryst. D* **2009**, *65*, 148-155.
(10) Structures were optimized using double-ζ basis set (def2-SVPP), all geometries were confirmed to be local minima (Int) or first order saddle points (for transition states, TS) on the potential energy surface by harmonic vibration frequency and intrinsic reaction coordinate calculation on the same level of theory, followed by single point calculations.
(11) Bannwarth, C.; Caldeweyher, E.; Ehlert, S.; Hansen, A.; Pracht, P.; Seibert, J.; Spicher, S.; Grimme, S. Extended tight-binding quantum chemistry methods. *WIREs Computational Molecular Science* **2020**, *11* (2), e1493.
(12) Bannwarth, C.; Ehlert, S.; Grimme, S. GFN2-xTB-An Accurate and Broadly Parametrized Self-Consistent Tight-Binding Quantum Chemical Method with Multipole Electrostatics and Density-Dependent Dispersion Contributions. *J. Chem. Theory Comput.* **2019**, *15*, 1652–1671.
(13) Frisch, M. J.; Trucks, G. W.; Schlegel, H. B.; Scuseria, G. E.; Robb, M. A.; Cheeseman, J. R.; Scalmani, G.; Barone, V.; Petersson, G. A.; Nakatsuji, H.; et al., *Gaussian Inc.*; Wallingford CT, 2016.
(14) Becke, A. D. Density-functional exchange-energy approximation with correct asymptotic behavior. *Phys. Rev. A* **1988**, *38*, 3098-3100.
(15) Becke, A. D. Density-functional thermochemistry. III. The role of exact exchange. *J. Chem. Phys.* **1993**, *98* (7), 5648-5652.
(16) Perdew, J. P. Density-functional approximation for the correlation energy of the inhomogeneous electron gas. *Phys. Rev. B* **1986**, *34*, 7406.
(17) Vosko, S. H.; Wilk, L.; Nusair, M. Accurate spin-dependent electron liquid correlation energies for local spin density calculations: a critical analysis. *Can. J. Phys.* **1980**, *58*, 1200-1211.
(18) Lee, C.; Yang, W.; Parr, R. G. Development of the Colle-Salvetti correlation-energy formula into a functional of the electron density. *Phys. Rev. B* **1988**, *37*, 785-789.
(19) Miehlich, B.; Savin, A.; Stoll, H.; Preuss, H. Results obtained with the correlation energy density functionals of Becke and Lee, Yang and Parr. *Chem. Phys. Lett.* **1989**, *157*, 200-206.
(20) Grimme, S.; Antony, J.; Ehrlich, S.; Krieg, H. A consistent and accurate ab initio parametrization of density functional dispersion correction (DFT-D) for the 94 elements H-Pu. *J. Chem. Phys.* **2010**, *132* (15), 154104-154123.
(21) Grimme, S.; Ehrlich, S.; Goerigk, L. Effect of the damping function in dispersion corrected density functional theory. *J. Comput. Chem.* **2011**, *32*, 1456-1465.
(22) Weigend, F.; Ahlrichs, R. Balanced basis sets of split valence, triple zeta valence and quadruple zeta valence quality for H to Rn: Design and assessment of accuracy. *Phys. Chem. Chem. Phys.* **2005**, *7*, 3297-3305.

(23) Marenich, A. V.; Cramer, C. J.; Truhlar, D. G. Universal Solvation Model Based on Solute Electron Density and on a Continuum Model of the Solvent Defined by the Bulk Dielectric Constant and Atomic Surface Tensions. *J. Chem. Phys.* **2009**, *113*, 6378-6396.
(24) Neese, F. The ORCA program system. *Wiley Interdiscip. Rev.-Comput. Mol. Sci.* **2012**, *2*, 73-78.
(25) Neese, F.; Wennmohs, F.; Becker, U.; Riplinger, C. The ORCA quantum chemistry program package. *J. Chem. Phys.* **2020**, *152*, 224108-224125.
(26) Neese, F. Software update: The ORCA program system - Version 5.0. *Wiley Interdiscip. Rev.-Comput. Mol. Sci.* **2022**, e1606-e1621.
(27) Riplinger, C.; Neese, F. An Efficient and Near Linear Scaling Pair Natural Orbital Based Local Coupled Cluster Method. *J. Chem. Phys.* **2013**, *138*, 034106.
(28) Riplinger, C.; Sandhoefer, B.; Hansen, A.; Neese, F. Natural Triple Excitations in Local Coupled Cluster Calculations With Pair Natural Orbitals. *J. Chem. Phys.* **2013**, *139*, 134101.
(29) Riplinger, C.; Pinski, P.; Becker, U.; Valeev, E. F.; Neese, F. Sparse Maps A Systematic Infrastructure for Reduced-Scaling Electronic Structure Methods. II. Linear Scaling Domain Based Pair Natural Orbital Coupled Cluster Theory. *J. Chem. Phys.* **2016**, *144*, 024109.
(30) Saitow, M.; Becker, U.; Riplinger, C.; Valeev, E. F.; Neese, F. A New Near-Linear Scaling, Efficient and Accurate, Open-Shell Domain-Based Local Pair Natural Orbital Coupled Cluster Singles and Doubles Theory. *J. Chem. Phys.* **2017**, *146*, 164105.
(31) Guo, Y.; Riplinger, C.; Becker, U.; Liakos, D. G.; Minenkov, Y.; Cavallo, L.; Neese, F. Communication: An Improved Linear Scaling Perturbative Triples Correction for the Domain Based Local Pair-Natural Orbital Based Singles and Doubles Coupled Cluster Method [DLPNO-CCSD(T)]. *J. Chem. Phys.* **2018**, *148*, 011101.
(32) Wang, Z., *EnePro version1.6* 2021.
(33) Becke, A. D.; Edgecombe, E. A simple measure of electron localization in atomic and molecular systems. *J. Chem. Phys.* **1990**, *92* (9), 5397–5403.
(34) Bader, R. F. W. A Quantum Theory of Molecular Structure and Its Applications. *Chem. Rev.* **1991**, *91*, 893-928.
(35) Wiberg, K. B. APPLICATION OF THE POPLE-SANTRY-SEGAL CNDO METHOD TO THE CYCLOPROPYLCARBINYL AND CYCLOBUTYL CATION AND TO BICYCLOBUTANE. *Tetrahedron* **1967**, *24*, 1083-1096.
(36) Glendening, E. D.; Badenhoop, J. K.; Reed, A. E.; Carpenter, J. E.; Bohmann, J. A.; Morales, C. M.; Landis, C. R.; Weinhold, F. NBO 6.0. *Theor. Chem.* **2013**.
(37) Weinhold, F.; Landis, C. R. *Valency and Bonding. A Natural Bond Orbital Donor-Acceptor Perspective,* ; Cambridge University Press, 2005.
(38) Weinhold, F.; Carpenter, J. E. *The Structure of Small Molecules and Ions*; Plenum Press, 1988.
(39) Carpenter, J. E.; Weinhold, F. *J. Mol. Struct.: THEOCHEM* **1988**, *169*, 41–62.
